# Using non-parametric Bayes shrinkage to assess relationships between multiple environmental and social stressors and neonatal size and body composition in the Healthy Start cohort

**DOI:** 10.1186/s12940-022-00934-z

**Published:** 2022-11-19

**Authors:** Sheena E. Martenies, Lauren Hoskovec, Ander Wilson, Brianna F. Moore, Anne P. Starling, William B. Allshouse, John L. Adgate, Dana Dabelea, Sheryl Magzamen

**Affiliations:** 1 Department of Kinesiology and Community Health, University of Illinois at Urbana-Champaign, 906 S Goodwin Ave, M/C 052, Urbana, IL 61801 USA; 2 Division of Nutritional Sciences, University of Illinois at Urbana-Champaign, Urbana, IL USA; 3 Department of Environmental and Radiological Health Sciences, Colorado State University, Fort Collins, CO USA; 4 Department of Statistics, Colorado State University, Fort Collins, CO USA; 5 Department of Epidemiology, Colorado School of Public Health, University of Colorado Anschutz Medical Campus, Aurora, CO USA; 6 Lifecourse Epidemiology of Adiposity and Diabetes (LEAD Center), University of Colorado Anschutz Medical Campus, Aurora, CO USA; 7 Department of Epidemiology, Gillings School of Global Public Health, University of North Carolina, Chapel Hill, NC USA; 8 Department of Environmental and Occupational Health, Colorado School of Public Health, University of Colorado Anschutz Campus, Aurora, CO USA; 9 Department of Pediatrics, School of Medicine, University of Colorado Anschutz Medical Campus, Aurora, CO USA

**Keywords:** Birth weight, Adiposity, Environmental mixtures, Social stressors, Air displacement plethysmography, Non-parametric Bayes shrinkage

## Abstract

**Background:**

Both environmental and social factors have been linked to birth weight and adiposity at birth, but few studies consider the effects of exposure mixtures. Our objective was to identify which components of a mixture of neighborhood-level environmental and social exposures were driving associations with birth weight and adiposity at birth in the Healthy Start cohort.

**Methods:**

Exposures were assessed at the census tract level and included air pollution, built environment characteristics, and socioeconomic status. Prenatal exposures were assigned based on address at enrollment. Birth weight was measured at delivery and adiposity was measured using air displacement plethysmography within three days. We used non-parametric Bayes shrinkage (NPB) to identify exposures that were associated with our outcomes of interest. NPB models were compared to single-predictor linear regression. We also included generalized additive models (GAM) to assess nonlinear relationships. All regression models were adjusted for individual-level covariates, including maternal age, pre-pregnancy BMI, and smoking.

**Results:**

Results from NPB models showed most exposures were negatively associated with birth weight, though credible intervals were wide and generally contained zero. However, the NPB model identified an interaction between ozone and temperature on birth weight, and the GAM suggested potential non-linear relationships. For associations between ozone or temperature with birth weight, we observed effect modification by maternal race/ethnicity, where effects were stronger for mothers who identified as a race or ethnicity other than non-Hispanic White. No associations with adiposity at birth were observed.

**Conclusions:**

NPB identified prenatal exposures to ozone and temperature as predictors of birth weight, and mothers who identify as a race or ethnicity other than non-Hispanic White might be disproportionately impacted. However, NPB models may have limited applicability when non-linear effects are present. Future work should consider a two-stage approach where NPB is used to reduce dimensionality and alternative approaches examine non-linear effects.

**Supplementary Information:**

The online version contains supplementary material available at 10.1186/s12940-022-00934-z.

## Background

Birth weight and adiposity at birth are important neonatal indicators of infant, childhood, and long-term health. At the population level, seemingly small shifts in mean birth weight over the past few decades have been associated with population-level increases in the proportion of neonates born low birth weight (LBW; < 2500 g) or small for gestational age (SGA; below the 10^th^ percentile for each completed week of gestation) [1–3]. SGA and LBW babies are at higher risk of childhood obesity, asthma, delayed neurodevelopment, and metabolic disorders later in adulthood [4–10]. Similarly, adiposity at birth, which is a measure of the proportion of body mass that is fat mass, is a potentially important predictor of obesity and metabolic disease risk later in life [11, 12].

The existing literature demonstrates the ability of both individual-level and neighborhood-level exposures to influence birth weight and adiposity in the neonatal period. Individual-level factors include maternal characteristics such as age and race/ethnicity, maternal behaviors such as active smoking and physical activity, and metabolic factors such as obesity or gestational diabetes [13–15]. At the neighborhood level, both environmental and social factors have been linked to these neonatal outcomes. These factors include higher neighborhood deprivation index scores [16], low neighborhood affluence [17], poor quality built environments (e.g., higher rates of vacant buildings or proximity to major roadways) [18, 19], and higher ambient air pollutant exposures [20–24].

Although there is evidence to suggest that both social and environmental factors are associated with birth weight and adiposity, few studies have examined the combined effects of exposures to hazards in multiple domains (*i.e.,* physical, social, and chemical exposures). There is growing interest in understanding the joint effects of both environmental hazards and social stressors to understanding how total neighborhood contexts can impact early life outcomes [25, 26]. Although the underlying mechanisms have not been fully elucidated, there are several hypothesized pathways by which prenatal exposure to environmental hazards and social stressors might impact neonatal growth and development. For example, oxidative stress and inflammation are pathways common to both environmental hazards and social stressors [27, 28]. Alternatively, social stressors experienced during the prenatal period may modify the effects of environmental hazards [29–31]. Evidence from previous studies suggests there may be important interactions between neighborhood factors environmental and social hazards, and that the effect of these combined stressors on birth outcomes may be synergistic [32–35]. Additionally, neighborhood environmental and social conditions reflect the legacy of structural racism in the United States perpetuated by redlining and racial segregation [36–39]. Thus, investigating the combined effects of neighborhood level factors that influence health represents an important opportunity to address longstanding health disparities.

We previously tested associations between neonatal outcomes combined prenatal factors at the neighborhood level in the Healthy Start cohort using an index-based exposure assessment method [40]. We reported lower birth weights and lower adiposity for infants born to mothers with higher combined exposure index scores after controlling for key individual-level risk factors. Our index-based approach provided information on where public health interventions might be most effective for improving neonatal outcomes. However, the use of an index did not allow us to identify which exposures or combination of exposures were driving these associations and thus identify the underlying etiologic agent. Alternative approaches that accommodate large numbers of exposure variables and potential interactions between these exposures are needed to better characterize these associations and identify targets for future public health interventions [41].

Using data from the Healthy Start cohort, we expanded upon our previous study by employing a non-parametric Bayes shrinkage (NPB) approach to examine linear associations between our neighborhood-level determinants and neonatal outcomes. For this data set, we needed to identify a statistical method that would be capable of handling variable selection among potentially highly correlated exposures within the context of interactions between exposures and between exposures and covariates. NPB is designed to facilitate selection among a large number of correlated predictors in a single model, thus having an advantage over traditional multipollutant models [42, 43]. We compared the results of our NPB model to single-exposure models (linear regression models). Because NPB is a linear modeling framework, we also utilized generalized additive models to explore potential non-linear exposure–response relationships. Based on the results of our previous study [40], we hypothesized that specific social exposures (*e.g.,* neighborhood level socioeconomic status) within the prenatal exposure mixture measured were driving associations with lower birth weight and adiposity.

## Methods

### Study population and study area

We used data from the ongoing Healthy Start pre-birth cohort study for this analysis. Details on the Healthy Start cohort are available elsewhere [44]. Briefly, pregnant women aged 16 or older expecting singleton births were recruited from the University of Colorado Hospital outpatient obstetrics clinics between 2009 and 2014. Two prenatal study visits that included a physical exam and questionnaires were conducted at median 17- and 27-weeks of gestation; additional data were abstracted from medical records. Our analysis did not exclude infants based on their term birth status (*i.e.,* we included pre-, full- and post-term births). All mother–child dyads who had an address at enrollment within the study area were eligible for this analysis. The Healthy Start study protocol was approved by the Colorado Multiple Institutional Review Board.

Although the Healthy Start study originally recruited participants from the nine county Denver metropolitan area, the study area for this analysis consisted of most census tracts within a smaller three county area (Adams, Arapahoe, and Denver counties). These counties were included due to the availability of exposure data (described next). We excluded the two eastern most census tracts within these counties because they were rural and not considered part of the metropolitan area. Census tracts were chosen to represent neighborhoods as they were the smallest spatial unit for which reliable exposure data were available.

### Exposure assessment

We selected our environmental and social exposures based on the indicators used by the CalEnviroScreen tool [45, 46] with some additional area-specific variables to reflect sources relevant to Denver, CO. Because our research question focused on the neighborhood context, all exposure variables were assessed at the census tract level and assigned to participants based on their address at the time of enrollment into the Healthy Start study. We briefly describe our exposure variables below. Additional details on our exposure variables are available elsewhere [40].

#### Ambient air pollution and temperature

We included two measures of ambient air pollution, fine particulate matter (PM_2.5_) and ozone (O_3_) and a variable for temperature. Monitoring data collected between 2009 and 2014 were obtained from the US Environmental Protection Agency [47]. Daily measurements at each monitor were averaged to biweekly concentrations. For PM_2.5_ and temperature we used the mean of all 24-h measurements and for O_3_ we used the mean of all daily 8-h maximum concentrations recorded during the two-week period. We used ordinary kriging to estimate biweekly measurements at each census tract centroid. Biweekly air pollutant and temperature exposures were then assigned to each participant based on the address at enrollment and the timing of conception and delivery and averaged over the duration of the pregnancy.

#### Built environment characteristics

We included nine exposure variables related to the built environment. The temporal resolution for these data was usually one year; we assumed minimal change in these variables between the start and end of our study period (2009–2014) These included: the mean percent tree cover and mean percent impervious surface within the census tract from the 2011 National Land Cover Database [48]; the mean annual average daily traffic (AADT) of road segments within the census tract from the Highway Performance Monitoring System [49]; and the minimum distance (in km) from the census tract centroid to hazardous land use sites, including Toxic Release Inventory sites, National Priority List sites, waste management sites, major emitters of criteria pollutants, confined animal feeding operations, and oil and gas wells [50–52].

#### Social stressors

Social stressor variables were intended to capture socioeconomic status (SES), demographic trends, and susceptibility at the neighborhood level. We included five variables from the 2010–2014 American Community Survey: the percentage of the population with less than a high school diploma, that was unemployed, and that identified as a race/ethnicity other than non-Hispanic white and the percentage of household speaking limited English or in poverty [53]. Crime incidence data were available from the Inter-university Consortium for Political and Social Research and the Denver Open Data Catalog [54, 55]. We used these incidence data to calculate 5-year rates of violent crimes and property crimes for each census tract. Data to calculate census-tract level hospitalization rates for respiratory and cardiovascular diseases came from the Colorado Hospital Association.

### Outcomes

Our two outcomes of interest were birth weight and adiposity at birth. Birth weight was measured at the time of delivery or was abstracted from medical records. Fat mass and fat-free (lean) body mass were measured within three days of birth using air displacement plethysmography (PEA POD, COSMED, Rome, Italy). Additional details on the PEA POD measurements for the Healthy Start cohort are available elsewhere [44]. We calculated adiposity (% fat mass) as fat mass divided by total body mass [56].

### Covariates

We selected our model covariates a priori by considering those included in our previous study [40] and other studies investigating the effect of neighborhood-level exposures on neonatal outcomes [16–18, 57–59]. Final covariate selection was informed by a directed acyclic graph. We included: maternal race/ethnicity, pre-pregnancy body mass index (BMI), and active smoking and secondhand smoke exposure. We excluded alcohol consumption because previous work in this cohort found levels were generally low among cohort participants and not associated with infant body composition measures [60, 61]. Consistent with our prior work and that of others [40, 62], maternal educational attainment was included as a proxy for family-level socioeconomic status (SES) because it is a consistent and reliable predictor of health outcomes for women and children [63, 64] and tends to be a more stable proxy for SES over time compared to income [65, 66]. We also included two measures of stress and depressive symptoms, Cohen’s Perceived Stress Scale (CPSS) and the Edinburgh Postnatal Depression Scale (EPDS), which were administered to mothers during their prenatal visits. Infant covariates included year of conception and sex. To address the potential for residual spatial autocorrelation, we included the longitude, latitude, and an interaction term (longitude × latitude) for the participant residential location. To account for temporal trends, we included the season of conception. For the adiposity models, we also included the number of days between delivery and PEA POD measurements. We did not adjust for gestational age in our models because our directed acyclic graph indicated gestational age was on the causal pathway.

Because individual-level race or ethnicity may modify the relationships between exposures and neonatal outcomes [67], we conducted sensitivity analyses where we stratified our results by maternal race/ethnicity (non-Hispanic White (NHW) vs. all other race and ethnic categories).

### Statistical analysis

All analyses were performed in R version 4.0.3 [68]. Prior to model fitting, exposures, outcomes, and covariates were summarized as means and standard deviations (SD) or frequencies as appropriate. To examine the potential for selection bias we assessed differences between included and excluded participants using chi square tests for categorical variables and t-tests for continuous variables. Categorical covariates were converted to indicator variables. To account for differences in units across the exposure variables and covariates, continuous variables were scaled prior to fitting the regression models.

#### Non-parametric Bayes shrinkage model

Our goal for this paper was to select from a complex mixture of neighborhood-level factors exposures that were associated with birth weight and adiposity. Therefore, our primary analysis used NPB, which is a Bayesian statistical method that fits a linear model with main effects of all exposures and all pairwise interactions while implementing both variable selection and regularization [42]. Specifically, NPB applies a Dirichlet zero-spiked process prior to the regression coefficients for each exposure. The Dirichlet process prior has a point mass at 0, which allows the model to select out exposures that have little influence on the outcome. Exposures with similar effects on the outcome can be clustered and assigned the same regression coefficient in the model. This reduces the variance in the presence of high correlation between the exposures. A similar approach is applied to the interaction effects. Here we included two-way interaction terms between each of the exposure variables and between each exposure and covariate. All interaction terms were selected with an additional zero-spiked Dirichlet process.

Our NPB model has the following form:

1 $${y}_{I}|\beta ,\gamma ,\zeta ,\delta ,{\sigma }^{2} \sim N({y}_{o}+ {x}_{i}^{T}\beta +{z}_{i}^{T}\zeta + {w}_{i}^{T}\gamma , {\sigma }^{2})$$

where ${x}_{i}$ is a vector of exposures, ${z}_{i}$ is a vector of pairwise multiplicative interactions between the exposures or the exposures and the covariates, ${w}_{i}$ is a vector of covariates, and ${\sigma }^{2}$ is the error variance. We implemented our NPB model using the “mmpack” package in R [69]. Posterior means and 95% credible intervals for the regression coefficients are reported for a standard deviation increase in each exposure metric. Consistent with other studies, we used 0.5 as the threshold for which posterior inclusion probabilities (PIPs) were considered to be indicative of an important effect of the exposure on the health outcome [70, 71]. Additional details on fitting the NPB model, including a description of the priors, is included in the Supplemental Materials.

Because variable selection by the NPB model may depend on the specification of hyperparameters, we conducted a sensitivity analysis in which the α and β values of the Gamma distributions for hyperparameters ${\alpha }_{1}$ and ${\alpha }_{2}$(see Text S1 in the Supplemental Materials) were set to 0.5, 1, 2, and 4.

#### Single exposure linear regression models

To serve as a comparison to the NPB method, we fit separate linear regression models for each exposure-outcome pair. These linear models were adjusted for all relevant covariates listed in Covariates. Results from these linear regression models were reported for a standard deviation increase in each exposure.

#### Generalized additive model

In a supplemental analysis meant to assess potential non-linear effects of our NPB-selected exposures of interest on birth weight or adiposity, we fit separate generalized additive models (GAMs) for any variables selected with NPB based on a PIP > 0.5. We used a smoothed term with penalized splines for the continuous exposures identified in the NPB model. Models were adjusted for all covariates listed in Covariates. We also repeated our sensitivity analysis and stratified GAMs by maternal race and ethnicity as described in Covariates. GAMs were fit in R using the “mgcv” package [72] and visualized using the “mgcViz” package [73].

## Results

### Study population

Our analytic cohort consisted of 897 mother–child dyads enrolled in the Healthy Start study, representing 64% of the original study population (Table 1). Of the excluded participants, *n* = 51 were excluded because they were missing data on birth weight, *n* = 259 were excluded because they lived outside the three-county study area, *n* = 172 were excluded because they were missing data on smoking exposures, and *n* = 37 were excluded because they were missing data on infant sex. There were few differences between included and excluded study participants (Table 1). Although birth weights were similar on average, we observed slightly higher adiposity for excluded participants. Included participants were also less likely to be non-Hispanic white and had higher mean CPSS compared to excluded participants. We did not observe any other differences between included and excluded participants for any of the other covariates of interest in this study.

**Table 1:** Summary of outcomes and covariates for the study population

Variable		Full Cohort (*n* = 1410)	Included (*n* = 897)	Excluded (*n* = 513)	*P*-value^a^
Birth weight (g)	mean ± SD	3205 ± 538	3208 ± 527	3199 ± 559	0.776
Adiposity (% fat mass)^b^	mean ± SD	9.2 ± 4.0	9.0 ± 4.0	9.5 ± 3.9	0.028
Maternal Race/Ethnicity					0.003
Hispanic/Latina	n (%)	351 (25)	238 (27)	113 (22)	
White non-Hispanic	n (%)	751 (52)	445 (50)	306 (60)	
African American	n (%)	219 (16)	154 (17)	65 (13)	
Other	n (%)	89 (6)	60 (7)	29 (6)	
Maternal age (years)	mean ± SD	27.8 ± 6.2	27.6 ± 6.2	28 ± 6.2	0.254
Mean CPSS Score	mean ± SD	18.8 ± 3.1	18.6 ± 3.1	19.2 ± 3.1	0.002
Mean EPDS Score	mean ± SD	4.4 ± 3.4	4.3 ± 3.3	4.6 ± 3.7	0.125
Pre-pregnancy body mass index (kg/m^2^)			0.583		
Underweight (< 18.5)	n (%)	44 (3)	30 (3)	14 (3)	
Normal (18.5—25)	n (%)	727 (52)	453 (51)	274 (54)	
Overweight (25—30)	n (%)	355 (25)	235 (26)	120 (24)	
Obese (> 30)	n (%)	280 (20)	179 (20)	101 (20)	
Maternal education level					0.816
Less than high school	n (%)	204 (14)	137 (15)	67 (13)	
High school or GED	n (%)	259 (18)	166 (19)	93 (18)	
Some college/Associate's	n (%)	334 (24)	208 (23)	126 (25)	
Bachelor's Degree	n (%)	309 (22)	196 (22)	113 (22)	
Graduate Degree	n (%)	304 (22)	190 (21)	114 (22)	
Any smoking during pregnancy?					0.940
No	n (%)	1286 (91)	819 (91)	467 (91)	
Yes	n (%)	124 (9)	78 (9)	46 (9)	
Any SHS exposure during pregnancy?					0.662
No	n (%)	924 (75)	666 (74)	258 (76)	
Yes	n (%)	314 (25)	231 (26)	83 (24)	
Infant sex					0.508
Female	n (%)	646 (48)	438 (49)	208 (47)	
Male	n (%)	696 (52)	459 (51)	237 (53)	
Gestational age (weeks)	mean ± SD	39.2 ± 1.9	39.3 ± 1.8	39.1 ± 2.2	0.052
Term status					0.147
Pre-term (< 37 weeks)	n (%)	90 (7)	60 (7)	30 (6)	
Early Term (37 to < 39 weeks)	n (%)	331 (24)	200 (22)	131 (28)	
Full Term (39 to < 41 weeks)	n (%)	802 (59)	538 (60)	264 (57)	
Late Term (41 to < 42 weeks)	n (%)	126 (9)	87 (10)	39 (8)	
Post-term (> = 42 weeks)	n (%)	15 (1)	12 (1)	3 (1)	
Low birth weight (< 2500 g)	n (%)	104 (8)	67 (7)	37 (8)	0.805
Days from delivery to PEA POD (days)	mean ± SD	1.6 ± 2.4	1.6 ± 2.5	1.8 ± 2.3	0.235

*Abbreviations:* *CPSS* Cohen Perceived Stress Scale, *EPSD* Edinburgh Postnatal Depression Scale, *SHS* Secondhand smoke

^a^ Chi square test for categorical variables; Student’s t test for continuous variables

^b^ Defined as (fat mass/total body mass) × 100%. Sample sizes for the adiposity variable are *n* = 1141 for the full cohort, *n* = 786 for the included cohort and *n* = 357 for the excluded cohort

### Exposure assessment

Exposures to environmental and social factors varied across participants (Table 2). Coefficients of variation were generally high (> 25%) for most (74%) of the exposures. Variability was lowest for the air pollutant exposures (mean PM_2.5_ and mean O_3_) and mean temperature across pregnancy. Correlations between environmental exposures and between environmental and social exposures were generally moderate (Pearson’s coefficients ranged from -0.5 to 0.7). Correlations tended to be stronger between the social determinants of health (Pearson’s coefficients ranged from -0.1 to 0.9; Figure S1).

**Table 2:** Summary statistics for all environmental and social exposures considered in the nonparametric Bayesian shrinkage model (*n* = 897)

Variable	Units	Mean ± SD	Min	Median	Max	CV
Environmental exposures						
Mean PM_2.5_	µg/m^3^	7.5 ± 0.6	5.8	7.5	9.1	8%
Mean O_3_	ppb	48.0 ± 3.1	40.8	47.8	58.3	8%
Mean temperature	°F	52.2 ± 4.8	37.3	52.4	66.4	9%
Tree cover	%	6.3 ± 3.1	0.2	6.1	18.7	50%
Impervious surfaces	%	40.5 ± 13.3	0.3	42.5	82.4	33%
AADT	Vehicles/d-km^2^	10,344 ± 8203	21	9074	54,618	79%
Distance to TRI sites	km	3.9 ± 2.6	0.1	3.3	21.9	66%
Distance to NPL sites	km	5.5 ± 3.3	0.0	5.5	23.6	60%
Distance to waste management sites	km	5.2 ± 2.3	0.5	5.0	11.7	44%
Distance to major emitters	km	8.3 ± 3.2	0.3	8.7	21.0	38%
Distance to CAFOs	km	36.8 ± 6.8	7.1	37.4	55.1	18%
Distance to mines or wells	km	3.4 ± 2.1	0.1	3.1	10.5	62%
Social exposures						
CVD hospitalizations	n/10,000	244.0 ± 45.2	127.9	243.8	471.8	19%
Respiratory hospitalizations	n/10,000	165.2 ± 33.0	95.2	164.1	319.8	20%
Violent crimes	n/1,000	12.8 ± 6.3	0.6	15.3	81.8	49%
Property crimes	n/1,000	55.4 ± 36.0	10.6	55.9	472.7	65%
Less than HS diploma	%	16.5 ± 12.7	0.0	12.8	56.9	77%
Unemployment	%	9.7 ± 5	1.8	8.7	27.5	51%
Households in poverty	%	15.3 ± 10.9	0.0	13.9	79.0	71%
Households speaking limited English	%	8.3 ± 8.3	0.0	5.8	39.1	100%
Percent persons of color	%	54.3 ± 22.9	2.7	54.6	93.7	42%

*Abbreviations:* *AADT* Annual average daily traffic, *CAFO* Confined animal feeding operation, *CV* Coefficient of variation, *CVD* Cardiovascular disease, *HS* High school, *NPL* National priorities list (Superfund sites), *O*_*3*_ Ozone, *PM*_*2.5*_ fine particulate matter, *TRI* Toxic release inventory

### Regression results

#### Nonparametric Bayes shrinkage

The NPB model, which included all exposures of interest in the same model, suggested that few of our exposures were strongly associated with birth weight (Table 3) based on our criterion of PIP = 0.5. Credible intervals for the posterior means were generally wide and contained zero. Only one variable had a PIP above 0.5 and a credible interval that did not contain zero: the interaction between mean ambient ozone concentration and temperature across pregnancy. A 3.1 ppb increase in mean O_3_ assessed across the pregnancy was associated with a 6.4 g decrease in birth weight (95% credible interval: -65.1 g to 6.9 g) and a 4.8 °F increase in mean temperature across pregnancy was associated with a 13.1 g increase (95% credible interval: -18.6 g to 189.3 g). For every 1 SD increase in temperature, the decrease in birth weight for a 1 SD increase in ozone was 162.0 g (95% CI: -242.3 g to -117.8 g) greater than expected if effects of the two exposures were additive. A plot showing the relationship between predicted and observed birth weight is included in the Supplemental Materials (Figure S2).

**Table 3:** Posterior means, 95% credible intervals, and posterior inclusion probabilities (PIPs) for each census tract level exposure regression coefficient in the NPB model, adjusted for all individual-level covariates. Results are presented for a 1 SD increase in each exposure

Variable	SD^c^	Birth Weight (g)		Adiposity (% Fat Mass)	
		Posterior Mean^a^ (95% CI)	PIP	Posterior Mean^a,b^ (95% CI)	PIP
Environmental Exposures					
Mean PM_2.5_ (μg/m^3^)	0.6	-2.4 (-29.3, 8.5)	0.31	-0.01 (-0.16, 0.11)	0.32
Mean O_3_ (ppb)	3.1	-6.4 (-65.1, 6.9)	0.37	-0.02 (-0.24, 0.09)	0.36
Mean temperature (°F)	4.8	13.1 (-18.6, 189.3)	0.38	-0.01 (-0.20, 0.12)	0.32
Mean O_3_ × Mean temperature		-162.0 (-242.3, -117.8)	1.00	-0.01 (-0.09, 0.00)	0.04
Tree cover (%)	3.1	-0.4 (-12.1, 11.6)	0.25	0.00 (-0.15, 0.11)	0.28
Impervious surfaces (%)	13.3	-0.7 (-14.4, 7.4)	0.25	-0.01 (-0.15, 0.09)	0.29
AADT (vehicles per day-km^2^)	8203	0.7 (-9, 18.2)	0.24	0.01 (-0.08, 0.21)	0.28
Distance to TRI sites (km)	2.6	-1.1 (-18.9, 8.9)	0.27	-0.01 (-0.17, 0.11)	0.32
Distance to NPL sites (km)	3.3	0.0 (-11.7, 14.6)	0.26	0.00 (-0.13, 0.14)	0.30
Distance to waste sites (km)	2.3	3.0 (-7.4, 39.2)	0.31	0.03 (-0.07, 0.34)	0.33
Distance to major emitters (km)	3.2	0.0 (-10.7, 13.1)	0.24	0.01 (-0.09, 0.19)	0.29
Distance to CAFOs (km)	6.8	-2.1 (-31, 14.2)	0.34	-0.02 (-0.24, 0.14)	0.35
Distance to mines or wells (km)	2.1	-1.7 (-22.6, 8.9)	0.31	-0.03 (-0.27, 0.07)	0.38
Social Exposures					
CVD hospitalizations (n per 10,000)	45.2	-1.4 (-18.4, 5.9)	0.28	-0.02 (-0.22, 0.08)	0.35
Resp. hospitalizations (n per 10,000)	33.0	-2.5 (-26.8, 5.0)	0.30	-0.02 (-0.21, 0.05)	0.35
Violent crimes (n per 1,000)	6.3	-0.3 (-12.2, 9.2)	0.25	-0.01 (-0.16, 0.09)	0.32
Property crimes (n per 1,000)	36.0	-1.5 (-18.3, 5.2)	0.28	-0.06 (-0.35, 0.03)	0.49
Less than HS diploma (%)	12.7	-1.1 (-17.9, 10.6)	0.29	-0.01 (-0.20, 0.09)	0.32
Unemployment (%)	5.0	-6.6 (-51.3, 2.5)	0.40	-0.03 (-0.30, 0.05)	0.37
Households speaking limited English (%)	10.9	-1 (-16.2, 6.9)	0.25	0.00 (-0.12, 0.19)	0.31
Households in poverty (%)	8.3	-1.1 (-15, 6.6)	0.26	-0.02 (-0.22, 0.06)	0.33
Persons of color (%)	22.9	-0.5 (-13.1, 12.6)	0.26	0.00 (-0.13, 0.15)	0.29

^a^ Models are adjusted for: maternal race/ethnicity, maternal educational attainment, maternal pre-pregnancy BMI, maternal age at delivery, maternal smoking during pregnancy, second-hand smoke exposure during pregnancy, mean perceived stress scale score across pregnancy, mean postnatal depression score across pregnancy, season of conception, year of conception, longitude, latitude, and the interaction between longitude and latitude

^b^ Models of adiposity are also adjusted for the number of days between delivery and PEA POD measurements

^c^ Effect estimates are reported for a 1 standard deviation increase in each exposure variable

None of the exposures considered in the NPB model for adiposity had credible interval that did not contain zero nor a PIP greater than the 0.5 threshold (Table 3). In general, we did not observe relationships between our exposures of interest and adiposity at birth. A plot showing the relationship between predicted and observed adiposity is included in the Supplemental Materials (Figure S3).

Neither the birth weight nor the adiposity model was sensitive to changes in the gamma distribution parameters for the mass concentration hyperparameter (Tables S2 and S3). For the four sensitivity analyses conducted, no differences in variables selected by the NPB model were observed.

We observed evidence of effect modification by maternal race/ethnicity in our NPB models for birth weight (Table 4). When stratifying by maternal race/ethnicity, we only observed an effect of temperature and ozone for participants who identified as a race or ethnicity other than NHW. For mothers who did not identify as NHW, for every 1 SD increase in temperature, the decrease in birth weight for a 1 SD increase in ozone was 206.2 g (95% CI: -258.3 g to -156.1 g) greater than expected if effects of the two exposures were additive.

**Table 4:** NPB models for birth weight stratified by maternal race/ethnicity (non-Hispanic White vs. all other race/ethnicity groups). Posterior means, 95% credible intervals, and posterior inclusion probabilities (PIPs) for each census tract level exposure regression coefficient in the NPB model. The model is adjusted for all individual-level covariates except race/ethnicity.^a^ Results are presented for a 1 SD increase in each exposure

Variable	Non-Hispanic White (*n* = 445)		All other Race/Ethnicity (*n* = 452)	
	Posterior Mean^b^ (95% CI)	PIP	Posterior Mean^b^ (95% CI)	PIP
Environmental Exposures				
Mean PM_2.5_ (μg/m^3^)	1.3 (-14.1, 27.8)	0.32	-15 (-94.3, 13.1)	0.45
Mean O_3_ (ppb)	-1.3 (-23.4, 17.6)	0.35	-15.5 (-103.5, 11.8)	0.43
Mean temperature (°F)	-1.1 (-22, 14.5)	0.33	0.9 (-36.2, 43.8)	0.32
Mean O_3_ × Mean temperature	-3.6 (-71.4, 0.0)	0.06	-206.2 (-258.3, -156.1)	1.00
Tree cover (%)	-0.1 (-15.3, 17.5)	0.31	1.2 (-19.9, 31.3)	0.27
Impervious surfaces (%)	-1.3 (-20.4, 10.9)	0.31	-1 (-29.4, 19.6)	0.25
AADT (vehicles per day-km^2^)	-0.9 (-18.7, 11.9)	0.30	3.3 (-10.5, 38.2)	0.28
Distance to TRI sites (km)	-2.3 (-26, 9.7)	0.35	3.5 (-15.3, 43.9)	0.30
Distance to NPL sites (km)	0.0 (-14.8, 20.7)	0.29	0.9 (-28.8, 32)	0.29
Distance to waste sites (km)	1.6 (-12.1, 31.9)	0.33	8.4 (-9.9, 65.4)	0.38
Distance to major emitters (km)	-1.0 (-17.8, 10.2)	0.31	7.8 (-8.7, 63.2)	0.35
Distance to CAFOs (km)	-0.3 (-22, 24.8)	0.35	-5.4 (-83.8, 36)	0.36
Distance to mines or wells (km)	-1.8 (-24.1, 12.2)	0.35	-0.4 (-33.9, 28.7)	0.29
Social Exposures				
CVD hospitalizations (n per 10,000)	-1 (-19.1, 13.4)	0.32	-2.5 (-47.8, 27.3)	0.32
Resp. hospitalizations (n per 10,000)	-0.8 (-17.8, 12.1)	0.30	-16.4 (-93.6, 6.3)	0.45
Violent crimes (n per 1,000)	-1.1 (-18.7, 11.5)	0.31	4.5 (-10.4, 48.6)	0.30
Property crimes (n per 1,000)	-1.9 (-22.2, 9.7)	0.32	1.9 (-15.1, 31.4)	0.26
Less than HS diploma (%)	-2.8 (-30.6, 6.9)	0.33	1.5 (-22.2, 36.4)	0.28
Unemployment (%)	-2.9 (-29.3, 6.7)	0.34	-12.3 (-80.2, 7.3)	0.41
Households speaking limited English (%)	-0.7 (-16.3, 12.4)	0.30	-2.2 (-39.8, 19.4)	0.27
Households in poverty (%)	-2.4 (-27.4, 8.0)	0.33	1.3 (-21.5, 34.5)	0.26
Persons of color (%)	-0.6 (-16.5, 12.3)	0.29	1.6 (-19.4, 35.5)	0.28

^a^ Models are adjusted for: maternal educational attainment, maternal pre-pregnancy BMI, maternal age at delivery, maternal smoking during pregnancy, second-hand smoke exposure during pregnancy, mean perceived stress scale score across pregnancy, mean postnatal depression score across pregnancy, season of conception, and year of conception

^b^ Effect estimates are reported for a 1 standard deviation increase in each exposure variable

#### Linear models

Following traditional epidemiological approaches, we also examined linear relationships between each individual exposure variable and our outcomes of interest. Of the 21 exposure variables included in our study, most (*n* = 14; 67%) were associated with lower birth weight, though most confidence intervals for the coefficients were wide and contained zero (Table S1). The strongest associations were seen for the percentage of adults in the census tract who were unemployed (β = -40.9 g, 95% CI: -76.8 g to -4.9 g) and the distance (km) to the nearest waste site (β = 39.7 g, 95% CI: 4.1 g to 75.4 g).

For adiposity (% fat mass), the linear regression models showed similar lack of association with most of the exposures of interest (Table S1). The strongest effect on % fat mass was observed for the rate of property crimes at the census tract level (β = -0.31 percentage points, 95% CI: -0.59 g to -0.04 percentage points) and the distance to waste sites (β = 0.36 percentage points, 95% CI: 0.07 to 0.66 percentage points). For most exposures, the confidence intervals were wide and contained 0.

#### Generalized additive models

**Fig. 1:**
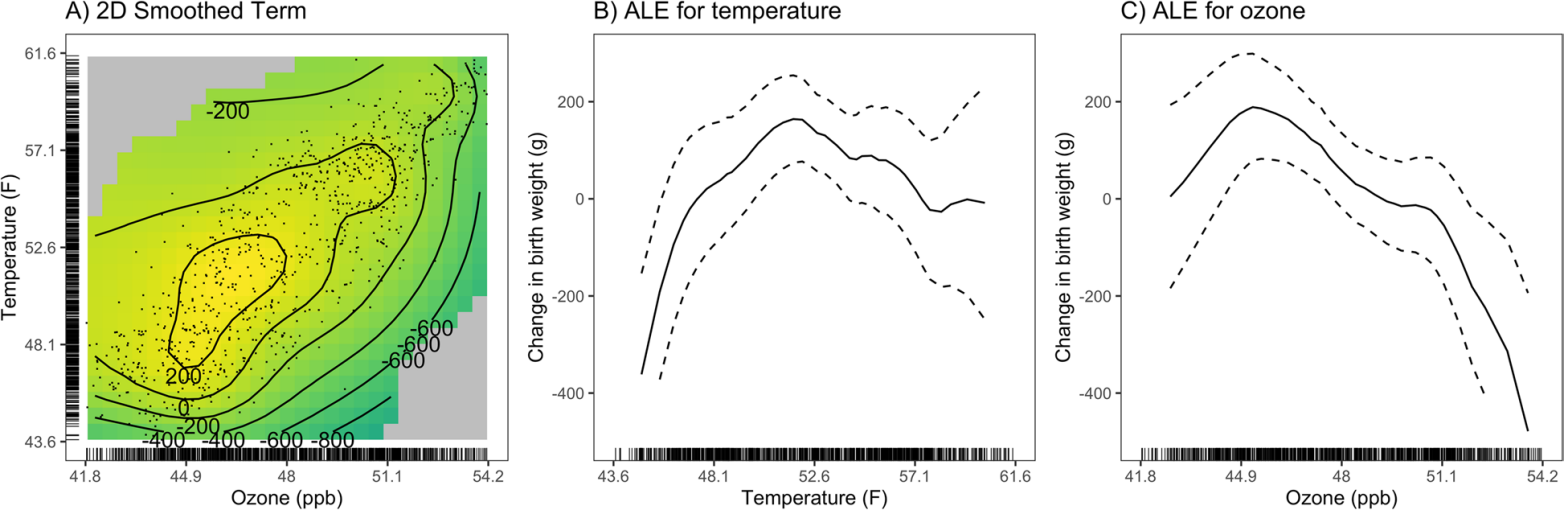
Exposure response curve for the 2D smoothed term for ozone and temperature in the generalized additive model (**A**) and accumulated local effects (ALE) plots showing the effect of temperature (**B**) and ozone (**C**) on birth weight. Note: axes are restricted to the middle 95% of observed ozone and temperature values

We examined potential non-linear effects for ozone and temperature using a GAM, adjusting for the same covariates included in the main NPB model (Fig. 1). To avoid overinterpreting effects at the margins, we restricted the axes to show the middle 95% of O_3_ and temperature observations. Plots showing the full range of data are available in the SDC (Figure S4). The exposure–response curve for the smoothed O_3_ and temperature term suggested a non-linear association between these exposures and birth weight (Fig. 1A). The accumulated local effect (ALE) plot for temperature showed an inverted-U shaped exposure–response relationship (Fig. 1B); the non-linear effect for ozone was less evident, with the ALE plot suggesting a more linear effect (Fig. 1C). The plots shown in Fig. 1 also suggest that the effect of temperature on birth weight is stronger than the effect of O_3_. We conducted a sensitivity analysis fitting the GAM with a dataset restricted to the middle 95% of O_3_ and temperature observations (Figure S5). These plots showed similar trends as those in Fig. 1.

**Fig. 2:**
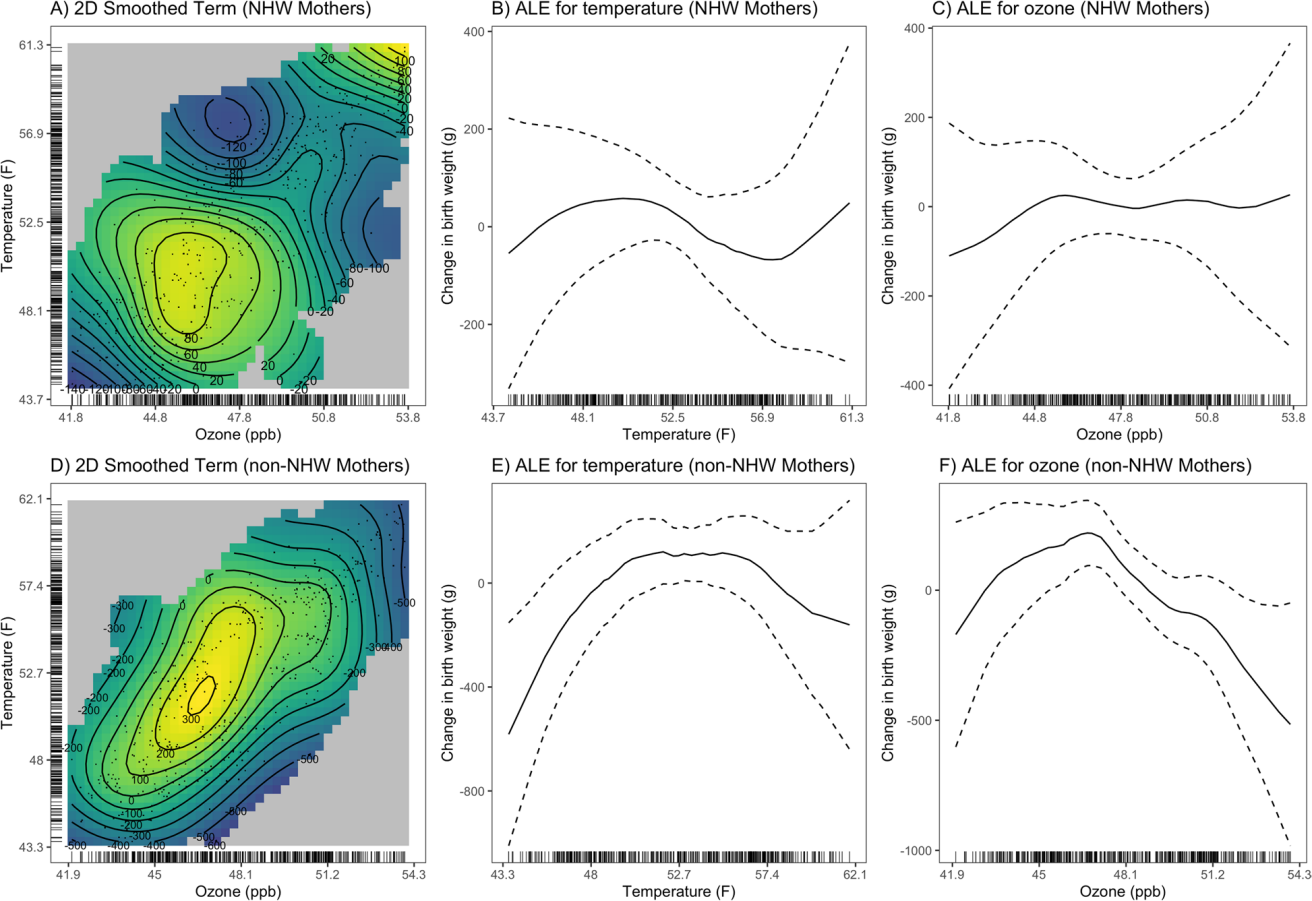
GAMs stratified by maternal race/ethnicity and restricted to the middle 95% of ozone and temperature observations. Plots show the exposure–response curve for the 2D smoothed term for ozone and temperature in the generalized additive model (**A**) and accumulated local effects (ALE) plots showing the effect of temperature (**B**) and ozone (**C**) on birth weight for non-Hispanic White (NHW) mothers and the exposure response curve for the 2-D smoothed term for ozone and temperature in the generalized additive model (**D**) and ALE plots showing the effect of temperature (**E**) and ozone (**F**) on birth weight for mothers identifying as any race or ethnicity other than non-Hispanic White

Results from GAMs stratified by maternal race/ethnicity showed similar effect modification as observed for the NPB models (Fig. 2, Figure S6). For stratified models where extreme values were excluded (Fig. 2), we observed non-linear associations between temperature and ozone for mothers who identified as a race/ethnicity other than non-Hispanic White (Fig. 2D-F) but not for non-White mothers (Fig. 2A-C).

## Discussion

Here we leveraged a non-parametric Bayesian shrinkage approach to identify which components of a mixture of exposures were driving previously reported associations between an exposure index and lower birth weight among infants born in the Denver metropolitan area. Using our mixtures approach (NPB), we identified the interaction between mean O_3_ concentrations and mean temperatures across the entire pregnancy as an important predictor of birth weight. Additional analyses using a GAM indicated that the relationship between O_3_, temperature, and birth weight may be non-linear. Our NPB results differed from those of the linear regression models, which identified only distance to waste sites, property crime rates, and unemployment as associated with our outcomes of interest and were likely biased by confounding from other correlated exposures.

Our results differ in some ways from other studies reported in the literature. For example, we did not identify an association between PM_2.5_ and birth weight in our cohort, which contradicts several other studies (as reviewed by Sun et al*.*) [74]. Our inability to detect this association may be in part due to the exposure metric used or due to low PM_2.5_ exposures in the study area. Average and maximum PM_2.5_ exposures across pregnancy in our cohort (7.5 and 9.1 µg/m^3^, respectively) were below the National Ambient Air Quality Standard for PM_2.5_ (annual average of 12 µg/m^3^). A similar Healthy Start study relying on trimester-specific averages derived from monitoring data also reported no association between PM_2.5_ and birth weight or adiposity [75]. In general, intraurban gradients of PM_2.5_ tend to be relatively flat and regulatory monitors poorly reflect variability from local sources [76]. Other pollutants such as black carbon that better reflect local sources in the region and characterize intraurban gradients in concentration may be more useful in future studies [77].

Our results indicating an interaction between temperature and O_3_ on birth weight are more consistent with previous studies [78]. Although this association has been reported less frequently in the literature, O_3_ exposures have been associated with lower birth weight (measured continuously) or increased odds of low birth weight (< 2,500 g) [78, 79]. Associations between both higher and lower temperatures and lower birth weight have also been reported [80–83]. Both ozone and ambient temperature exposures have been linked to systemic inflammation in adults [84–88]. Thus, we hypothesize that our results showing a relationship between temperature and O_3_ on birth weight may be due to maternal systemic inflammation, which is known to influence fetal growth [89].

Importantly, our study provides additional evidence of effect modification by maternal race and ethnicity on associations between environmental hazards and child health outcomes. Associations between our exposures of interest and birth weight were stronger for mothers from historically minoritized race or ethnicity groups compared to non-Hispanic White mothers. Several studies in the past two decades have reported racial disparities in low birth weight, which is more likely as population mean birthweights shift downward, over the last few decades [90–92]. Relationships between race or ethnicity, neighborhood quality, and neonatal outcomes are complicated, but stress is a likely mediating factor [93].

There were important differences between the results of the single-exposure linear regression analysis and the results from the NPB model. In the linear regression models, higher exposures to social stressors tended to be associated with lower birth weights and adiposity in the linear regression models, though the confidence intervals for the regression coefficients tended to be wide and contain zero. These results are consistent with our previous index-based analysis which found that associations between a combined exposure index and birth weight were likely driven by the social stressor component of the score [40]. However, the NPB model identified the interaction between ozone and temperature as being an important predictor of birth weight. Neither temperature nor ozone were associated with birth weight in the linear regression models. Differences between results derived from the NPB model and the linear regression models may be due to residual confounding by co-exposures in the single-exposure linear regression models. These models also do not include the interaction term that was important in the NPB model. The exposures included in our data set are moderately to highly correlated, and a single-exposure model is likely not sufficient to address this correlation. Using NPB as a modeling approach allowed us to consider each of these exposures at once in a framework that allows correlated exposures to be assigned similar coefficients when effects cannot be separated [43].

Additionally, differences between our NPB model and our original index-based approach also suggest that our combined exposure index may have characterized factors in the neighborhood that are not fully explained by any one variable. Notably, temperature was not included in our original index. Our exposures were originally chosen to reflect the combination of factors often included in screening tools for environmental justice concerns, *e.g.,* CalEnviroScreen [45] or EJScreen [94]; thus, we may have captured trends in neighborhood quality rather than specific etiologic factors for lower birth weight and adiposity. Future mixtures-based approaches for birth weight should consider moving beyond the typical indicators of health and neighborhood quality and focus more on those exposures that are suspected of being causally linked to neonatal outcomes.

There are some important limitations to our approach that should be considered when interpreting these results. First, the census tract estimates of exposure generally have limited temporal resolution. Due to the limitations of publicly available data at the spatial resolution used here, we relied on annual or five-year average estimates for most of our exposures. Second, there is no universal definition for neighborhood, and census tracts may not be the most meaningful unit of analysis; boundaries that reflect policy or other jurisdictional boundaries (e.g., school districts or municipal boundaries) may be more appropriate for some exposures [95].Third, as discussed above, we chose our exposures based on existing screening tools rather than specific exposures known to be associated with birth weight and adiposity. Future work should consider how and for what purpose existing public health indices are developed and may develop separate indices for different types of health outcomes. Lastly, we focused on a single metropolitan area where the range of exposures is limited. Replication of these methods in multi-city studies should broaden the range of exposures and better elucidate the relationships between multiple environmental and social hazards and neonatal health.

Despite these limitations, our approach demonstrated the strengths of using NPB in a mixtures context. Our use of NPB allowed us to include a large number of correlated exposures, which would otherwise be a challenge in a multipollutant model due to the potential for multicollinearity and variance inflation [42]. In this context, NPB has advantages over the least absolute shrinkage and selection operator (LASSO) which is widely used for variable selection. In particular, LASSO tends to perform poorly when variables are highly correlated [96, 97]. Additionally, LASSO does not account for uncertainty in variable selection [42]. In contrast, NPB can cluster and assign equal regression coefficients to exposures, which reduces the effect of correlation among exposures and is a strength when it is difficult to separate the effects of the two exposures [43]. In future cases where more than two exposures are identified, methods like Bayesian Kernel Machine Regression (BKMR) may be useful. BKMR is a flexible approach for modeling the effects of exposure mixtures that is capable of identifying nonlinear main effects and interactions among mixture components [98], though it may not be well suited for selecting among a large number of predictors [43], Thus, a two-stage approach where NPB models reduce the number of candidate predictors and methods like BKMR explore the complex interactions between exposures of interest might be most appropriate.

Overall, our NPB model, which was designed to select among a large number of correlated exposures, identified a joint effect of O_3_ and temperature as important predictor of birth weight, and the GAM indicated these association may be non-linear. Our results suggest there is a potential role for neighborhood-level environmental and social stressors in the prenatal period to influence fetal growth, but that additional studies are needed to identify important drivers in this exposure mixture. Future work may consider imputing missing values for key covariates to increase the sample size available for more complex statistical approaches such as BKMR. Further, it will be crucial to examine non-linear dose response relationships when assessing the effects of joint exposures assessed at the neighborhood level. When non-linear relationships are anticipated, additional variable selection approaches could be considered [99].

## Supplementary Information

**Additional file 1.** Supplemental Materials.

## Data Availability

The analysis code used in this study are available on the primary author’s GitHub page (https://github.com/smartenies/ECHO_Aim1_CEI_Mixtures). The Healthy Start data set is not publicly available because it contains sensitive personal health information. Deidentified data from the Healthy Start study are available for analyses related to the aims of the study upon reasonable request.

## Abbreviations

AATD: Annual average daily traffic
ALE: Accumulated local effect
BKMR: Bayesian Kernel Machine Regression
BMI: Body mass index
CPSS: Cohen’s Perceived Stress Scale
EPDS: Edinburgh Postnatal Depression Scale
GAM: Generalized additive model
LBW: Low birth weight
NHW: Non-Hispanic White
NPB: Non-parametric Bayes shrinkage
O_3_: Ozone
PIP: Posterior inclusion probability
PM_2.5_: Particulate matter with an aerodynamic diameter less than 2.5 microns
SD: Standard deviation
SES: Socioeconomic status
SGA: Small for gestational age

## References

[1] Donahue SMA, Kleinman KP, Gillman MW, Oken E (2010). Trends in birth weight and gestational length among singleton term births in the United States. Obstet Gynecol.

[2] Morisaki N, Esplin MS, Varner MW, Henry E, Oken E (2013). Declines in birth weight and fetal growth independent of gestational length. Obstet Gynecol.

[3] Oken E. Secular trends in birthweight. Recent advances in growth research: nutritional, molecular and endocrine perspectives. 2013;71:103–14. 10.1159/000345196 23502160

[4] Ong KK, Loos RJF (2006). Rapid infancy weight gain and subsequent obesity: systematic reviews and hopeful suggestions. Acta Paediatr.

[5] Nam H-K, Lee K-H (2018). Small for gestational age and obesity: epidemiology and general risks. Ann Pediatr Endocrinol Metab.

[6] Longo S, Bollani L, Decembrino L, Di Comite A, Angelini M, Stronati M (2013). Short-term and long-term sequelae in intrauterine growth retardation (IUGR). J Matern Fetal Neonatal Med.

[7] van Wassenaer A (2005). Neurodevelopmental consequences of being born SGA. Pediatr Endocrinol Rev.

[8] Savchev S, Sanz-Cortes M, Cruz-Martinez R, Arranz A, Botet F, Gratacos E (2013). Neurodevelopmental outcome of full-term small-for-gestational-age infants with normal placental function. Ultrasound Obstet Gynecol.

[9] Xu X-F, Li Y-J, Sheng Y-J, Liu J-L, Tang L-F, Chen Z-M. Effect of low birth weight on childhood asthma: a meta-analysis. BMC Pediatr. 2014;14. Available from: https://www.ncbi.nlm.nih.gov/pmc/articles/PMC4288645/. Cited 2018 Sep 10. 10.1186/1471-2431-14-275 PMC4288645 25339063

[10] Jornayvaz FR, Vollenweider P, Bochud M, Mooser V, Waeber G, Marques-Vidal P. Low birth weight leads to obesity, diabetes and increased leptin levels in adults: the CoLaus study. Cardiovasc Diabetol. 2016;15. Available from: https://www.ncbi.nlm.nih.gov/pmc/articles/PMC4855501/. Cited 2018 May 17. 10.1186/s12933-016-0389-2 PMC4855501 27141948

[11] Demerath EW, Fields DA (2014). Body composition assessment in the infant. Am J Hum Biol.

[12] Moore BF, Harrall KK, Sauder KA, Glueck DH, Dabelea D. Neonatal adiposity and childhood obesity. Pediatrics. 2020;146:e20200737. 10.1542/peds.2020-0737 PMC7461209 32796097

[13] Valero de Bernabé J, Soriano T, Albaladejo R, Juarranz M, Calle ME, Martínez D (2004). Risk factors for low birth weight: a review. Eur J Obstet Gynecol Reprod Biol.

[14] Sewell MF, Huston-Presley L, Super DM, Catalano P (2006). Increased neonatal fat mass, not lean body mass, is associated with maternal obesity. Am J Obstet Gynecol.

[15] Harvey NC, Poole JR, Javaid MK, Dennison EM, Robinson S, Inskip HM (2007). Parental determinants of neonatal body composition. J Clin Endocrinol Metab.

[16] Vos AA, Posthumus AG, Bonsel GJ, Steegers EAP, Denktaş S (2014). Deprived neighborhoods and adverse perinatal outcome: a systematic review and meta-analysis. Acta Obstet Gynecol Scand.

[17] Kane JB, Miles G, Yourkavitch J, King K (2017). Neighborhood context and birth outcomes: Going beyond neighborhood disadvantage, incorporating affluence. SSM - Population Health.

[18] Nowak AL, Giurgescu C (2017). The built environment and birth outcomes: a systematic review. MCN Am J Matern Child Nurs.

[19] Woods N, Gilliland J, Seabrook JA (2017). The influence of the built environment on adverse birth outcomes. J Neonatal Perinatal Med.

[20] Chiu Y-HM, Hsu H-HL, Wilson A, Coull BA, Pendo MP, Baccarelli A (2017). Prenatal particulate air pollution exposure and body composition in urban preschool children: examining sensitive windows and sex-specific associations. Environ Res.

[21] Fioravanti S, Cesaroni G, Badaloni C, Michelozzi P, Forastiere F, Porta D (2018). Traffic-related air pollution and childhood obesity in an Italian birth cohort. Environ Res.

[22] Schembari A, de Hoogh K, Pedersen M, Dadvand P, Martinez D, Hoek G (2015). Ambient air pollution and newborn size and adiposity at birth: differences by maternal ethnicity (the Born in Bradford Study Cohort). Environ Health Perspect.

[23] Westergaard N, Gehring U, Slama R, Pedersen M (2017). Ambient air pollution and low birth weight - are some women more vulnerable than others?. Environ Int.

[24] Stieb DM, Chen L, Eshoul M, Judek S (2012). Ambient air pollution, birth weight and preterm birth: a systematic review and meta-analysis. Environ Res.

[25] Padula AM, Rivera-Núñez Z, Barrett ES (2020). Combined impacts of prenatal environmental exposures and psychosocial stress on offspring health: air pollution and metals. Curr Envir Health Rpt.

[26] Koman PD, Hogan KA, Sampson N, Mandell R, Coombe CM, Tetteh MM (2018). Examining joint effects of air pollution exposure and social determinants of health in defining “at-risk” populations under the clean air act: susceptibility of pregnant women to hypertensive disorders of pregnancy. World Med Health Policy.

[27] Rakers F, Rupprecht S, Dreiling M, Bergmeier C, Witte OW, Schwab M (2020). Transfer of maternal psychosocial stress to the fetus. Neurosci Biobehav Rev.

[28] Erickson AC, Arbour L (2014). The shared pathoetiological effects of particulate air pollution and the social environment on fetal-placental development. J Environ Public Health.

[29] Brunst KJ, Sanchez-Guerra M, Chiu Y-HM, Wilson A, Coull BA, Kloog I (2018). Prenatal particulate matter exposure and mitochondrial dysfunction at the maternal-fetal interface: effect modification by maternal lifetime trauma and child sex. Environ Int.

[30] Deguen S, Kihal-Talantikite W, Gilles M, Danzon A, Carayol M, Zmirou-Navier D (2021). Are the effects of air pollution on birth weight modified by infant sex and neighborhood socioeconomic deprivation? A multilevel analysis in Paris (France). PLoS ONE.

[31] Erickson AC, Ostry A, Chan LHM, Arbour L (2016). The reduction of birth weight by fine particulate matter and its modification by maternal and neighbourhood-level factors: a multilevel analysis in British Columbia. Canada Environ Health.

[32] Généreux M, Auger N, Goneau M, Daniel M (2008). Neighbourhood socioeconomic status, maternal education and adverse birth outcomes among mothers living near highways. J Epidemiol Community Health.

[33] Padula AM, Mortimer KM, Tager IB, Hammond SK, Lurmann FW, Yang W (2014). Traffic-related air pollution and risk of preterm birth in the San Joaquin Valley of California. Ann Epidemiol.

[34] Ponce NA, Hoggatt KJ, Wilhelm M, Ritz B (2005). Preterm birth: the interaction of traffic-related air pollution with economic hardship in Los Angeles neighborhoods. Am J Epidemiol.

[35] Yi O, Kim H, Ha E (2010). Does area level socioeconomic status modify the effects of PM10 on preterm delivery?. Environ Res.

[36] Bailey ZD, Feldman JM, Bassett MT (2021). How structural racism works — racist policies as a root cause of U.S. racial health inequities. N Engl J Med..

[37] Groos M, Wallace M, Hardeman R, Theall K. Measuring inequity: a systematic review of methods used to quantify structural racism. J Health Disparities Res Pract. 2018;11. Available from: https://digitalscholarship.unlv.edu/jhdrp/vol11/iss2/13

[38] Gutschow B, Gray B, Ragavan MI, Sheffield PE, Philipsborn RP, Jee SH (2021). The intersection of pediatrics, climate change, and structural racism: Ensuring health equity through climate justice. Curr Probl Pediatr Adolesc Health Care.

[39] Payne-Sturges DC, Gee GC, Cory-Slechta DA (2021). Confronting racism in environmental health sciences: moving the science forward for eliminating racial inequities. Environ Health Perspect.

[40] Martenies SE, Allshouse WB, Starling AP, Ringham BM, Glueck DH, Adgate JL (2019). Combined environmental and social exposures during pregnancy and associations with neonatal size and body composition: the Healthy Start study. Environ Epidemiol.

[41] Appleton AA, Holdsworth EA, Kubzansky LD (2016). A systematic review of the interplay between social determinants and environmental exposures for early-life outcomes. Curr Environ Health Rep.

[42] Herring AH (2010). Nonparametric bayes shrinkage for assessing exposures to mixtures subject to limits of detection. Epidemiology.

[43] Hoskovec L, Benka-Coker W, Severson R, Magzamen S, Wilson A (2021). Model choice for estimating the association between exposure to chemical mixtures and health outcomes: a simulation study. PLoS ONE.

[44] Harrod CS, Chasan-Taber L, Reynolds RM, Fingerlin TE, Glueck DH, Brinton JT (2014). Physical activity in pregnancy and neonatal body composition: the Healthy Start study. Obstet Gynecol.

[45] Cushing L, Faust J, August LM, Cendak R, Wieland W, Alexeeff G (2015). Racial/ethnic disparities in cumulative environmental health impacts in California: Evidence From a Statewide Environmental Justice Screening Tool (CalEnviroScreen 1.1). Am J Public Health.

[46] Office of Environmental Health Hazard Assessment. CalEnviroScreen 3.0. OEHHA. 2016. Available from: https://oehha.ca.gov/calenviroscreen/report/calenviroscreen-30. Cited 2017 Nov 28.

[47] US Environmental Protection Agency [US EPA] (2017). AQS data mart.

[48] Multi-Resolution Land Characteristics Consortium. (2017). National land cover database.

[49] US Department of Transportation [US DOT] (2018). Highway performance monitoring system.

[50] Colorado Department of Public Health and Environment [CDPHE] (2018). Maps and GIS for health and environment.

[51] Colorado Oil and Gas Conservation Commission [COGCC] (2018). Colorado oil and gas information system.

[52] US Environmental Protection Agency. 2011 National Emissions Inventory (NEI) Data. US EPA. 2015. Available from: https://www.epa.gov/air-emissions-inventories/2011-national-emissions-inventory-nei-data. Cited 2018 Aug 27.

[53] US Census Bureau (2014). 2010–2014 American Community Survey (ACS) 5-year estimates.

[54] Inter-university Consortium for Political and Social Research [ICPSR] (2018). Uniform Crime Reporting Program Data Series.

[55] City and County of Denver (2017). Denver Open Data Catalog.

[56] Starling AP, Brinton JT, Glueck DH, Shapiro AL, Harrod CS, Lynch AM (2015). Associations of maternal BMI and gestational weight gain with neonatal adiposity in the Healthy Start study. Am J Clin Nutr.

[57] Morello-Frosch R, Jesdale BM, Sadd JL, Pastor M (2010). Ambient air pollution exposure and full-term birth weight in California. Environ Health.

[58] Ncube CN, Enquobahrie DA, Albert SM, Herrick AL, Burke JG (2016). Association of neighborhood context with offspring risk of preterm birth and low birthweight: a systematic review and meta-analysis of population-based studies. Soc Sci Med.

[59] Shmool JLC, Bobb JF, Ito K, Elston B, Savitz DA, Ross Z (2015). Area-level socioeconomic deprivation, nitrogen dioxide exposure, and term birth weight in New York City. Environ Res.

[60] Shapiro ALB, Kaar JL, Crume TL, Starling AP, Siega-Riz AM, Ringham BM (2016). Maternal diet quality in pregnancy and neonatal adiposity: the Healthy Start study. Int J Obes (Lond).

[61] Sauder KA, Kaar JL, Starling AP, Ringham BM, Glueck DH, Dabelea D (2017). Predictors of infant body composition at 5 months of age: the Healthy Start study. J Pediatr.

[62] Dunlop AL, Essalmi AG, Alvalos L, Breton C, Camargo CA, Cowell WJ (2021). Racial and geographic variation in effects of maternal education and neighborhood-level measures of socioeconomic status on gestational age at birth: findings from the ECHO cohorts. PLoS ONE.

[63] Kramer Ms, Séguin L, Lydon J, Goulet L (2000). Socio-economic disparities in pregnancy outcome: why do the poor fare so poorly?. Paediatr Perinat Epidemiol.

[64] Parker JD, Schoendorf KC, Kiely JL (1994). Associations between measures of socioeconomic status and low birth weight, small for gestational age, and premature delivery in the United States. Ann Epidemiol.

[65] Metcalf P, Scragg R, Davis P (2007). Relationship of different measures of socioeconomic status with cardiovascular disease risk factors and lifestyle in a New Zealand workforce survey. N Z Med J.

[66] Herd P, Goesling B, House JS (2007). Socioeconomic position and health: the differential effects of education versus income on the onset versus progression of health problems. J Health Soc Behav.

[67] Jones CP (2001). Invited commentary: “race”, racism, and the practice of epidemiology. Am J Epidemiol.

[68] R Core Team (2020). R: A language and environment for statistical computing.

[69] Hoskovec L (2019). mmpack: Implement methods for multipollutant mixtures analyses.

[70] Coull BA, Bobb JF, Wellenius GA, Kioumourtzoglou MA, Mittleman MA, Koutrakis P, et al. Part 1. Statistical learning methods for the effects of multiple air pollution constituents. Res Rep Health Eff Inst. 2015;(183 Pt 1-2):5–50. 26333238

[71] Barbieri MM, Berger JO (2004). Optimal predictive model selection. Ann Stat Inst Math Stat.

[72] Wood SN (2011). Fast stable restricted maximum likelihood and marginal likelihood estimation of semiparametric generalized linear models. J Royal Statistical Soc Series B.

[73] Fasiolo M, Nedellec R, Goude Y, Wood SN. Scalable visualisation methods for modern generalized additive models. Archix preprint. 2018;10632.

[74] Sun X, Luo X, Zhao C, Zhang B, Tao J, Yang Z (2016). The associations between birth weight and exposure to fine particulate matter (PM2.5) and its chemical constituents during pregnancy: a meta-analysis. Environ Pollut.

[75] Starling AP, Moore BF, Thomas DSK, Peel JL, Zhang W, Adgate JL (2020). Prenatal exposure to traffic and ambient air pollution and infant weight and adiposity: the Healthy Start study. Environ Res.

[76] Matte TD, Ross Z, Kheirbek I, Eisl H, Johnson S, Gorczynski JE (2013). Monitoring intraurban spatial patterns of multiple combustion air pollutants in New York City: design and implementation. J Expos Sci Environ Epidemiol.

[77] Martenies SE, Keller JP, WeMott S, Kuiper G, Ross Z, Allshouse WB, et al. A spatiotemporal prediction model for black carbon in the Denver Metropolitan Area, 2009–2020. Environ Sci Technol. 2021. Available from: 10.1021/acs.est.0c06451. American Chemical Society; cited 2021 Feb 26. 10.1021/acs.est.0c06451 PMC8313050 33596061

[78] Bekkar B, Pacheco S, Basu R, DeNicola N (2020). Association of air pollution and heat exposure with preterm birth, low birth weight, and stillbirth in the US: a systematic review. JAMA Netw Open.

[79] Salam MT, Millstein J, Li Y-F, Lurmann FW, Margolis HG, Gilliland FD (2005). Birth outcomes and prenatal exposure to ozone, carbon monoxide, and particulate matter: results from the children’s health study. Environ Health Perspect.

[80] Chersich MF, Pham MD, Areal A, Haghighi MM, Manyuchi A, Swift CP (2020). Associations between high temperatures in pregnancy and risk of preterm birth, low birth weight, and stillbirths: systematic review and meta-analysis. BMJ.

[81] Li S, Wang J, Xu Z, Wang X, Xu G, Zhang J (2018). Exploring associations of maternal exposure to ambient temperature with duration of gestation and birth weight: a prospective study. BMC Pregnancy Childbirth.

[82] Sun S, Spangler KR, Weinberger KR, Yanosky JD, Braun JM, Wellenius GA. Ambient temperature and markers of fetal growth: a retrospective observational study of 29 million U.S. Singleton Births. Environ Health Perspect. 2019;127. Available from: https://ehp.niehs.nih.gov/doi/10.1289/EHP4648. Cited 2021 Apr 8. 10.1289/EHP4648 PMC6792370 31162981

[83] Lawlor DA, Leon DA, Smith GD (2005). The association of ambient outdoor temperature throughout pregnancy and offspring birthweight: findings from the Aberdeen Children of the 1950s cohort. BJOG.

[84] Arjomandi M, Wong H, Donde A, Frelinger J, Dalton S, Ching W (2015). Exposure to medium and high ambient levels of ozone causes adverse systemic inflammatory and cardiac autonomic effects. Am J Physiol Heart Circ Physiol.

[85] Balmes JR, Arjomandi M, Bromberg PA, Costantini MG, Dagincourt N, Hazucha MJ, et al. Ozone effects on blood biomarkers of systemic inflammation, oxidative stress, endothelial function, and thrombosis: the Multicenter Ozone Study in oldEr Subjects (MOSES). PLoS One. 2019;14. Available from: https://www.ncbi.nlm.nih.gov/pmc/articles/PMC6760801/. Cited 2020 Nov 9. 10.1371/journal.pone.0222601 PMC6760801 31553765

[86] Khafaie MA, Yajnik CS, Mojadam M, Khafaie B, Salvi SS, Ojha A, et al. Association between ambient temperature and blood biomarker of systemic inflammationin (C-reactive protien) in diabetes patients. Arch Med. 2016;8. Available from: https://www.archivesofmedicine.com/abstract/association-between-ambient-temperature-and-blood-biomarker-of-systemic-inflammationin-creactive-protien-in-diabetes-patients-9582.html. iMedPub; Cited 2021 Apr 8.

[87] Peters A, Panagiotakos D, Picciotto S, Katsouyanni K, Löwel H, Jacquemin B (2008). Air temperature and inflammatory responses in myocardial infarction survivors. Epidemiology.

[88] Halonen JI, Zanobetti A, Sparrow D, Vokonas PS, Schwartz J (2010). Associations between outdoor temperature and markers of inflammation: a cohort study. Environ Health.

[89] Dimasuay KG, Boeuf P, Powell TL, Jansson T. Placental responses to changes in the maternal environment determine fetal growth. Front Physiol. 2016;7. Available from: https://www.frontiersin.org/articles/10.3389/fphys.2016.00012/full. Frontiers; Cited 2021 Feb 17. 10.3389/fphys.2016.00012 PMC4731498 26858656

[90] Blumenshine P, Egerter S, Barclay CJ, Cubbin C, Braveman PA (2010). Socioeconomic disparities in adverse birth outcomes: a systematic review. Am J Prev Med.

[91] Grobman WA, Parker CB, Willinger M, Wing DA, Silver RM, Wapner RJ (2018). Racial disparities in adverse pregnancy outcomes and psychosocial stress. Obstet Gynecol.

[92] Lu MC, Halfon N (2003). Racial and ethnic disparities in birth outcomes: a life-course perspective. Matern Child Health J.

[93] Almeida J, Bécares L, Erbetta K, Bettegowda VR, Ahluwalia IB (2018). Racial/ethnic inequities in low birth weight and preterm birth: the role of multiple forms of stress. Matern Child Health J.

[94] US Environmental Protection Agency [US EPA] (2015). EJSCREEN.

[95] Riley AR (2018). Neighborhood Disadvantage, residential segregation, and beyond—lessons for studying structural racism and health. J Racial and Ethnic Health Disparities.

[96] Zou H, Hastie T (2005). Regularization and variable selection via the elastic net. J Royal Statistical Soc Series B.

[97] Barrera-Gómez J, Agier L, Portengen L, Chadeau-Hyam M, Giorgis-Allemand L, Siroux V (2017). A systematic comparison of statistical methods to detect interactions in exposome-health associations. Environ Health.

[98] Bobb JF, Valeri L, Claus Henn B, Christiani DC, Wright RO, Mazumdar M (2015). Bayesian kernel machine regression for estimating the health effects of multi-pollutant mixtures. Biostatistics.

[99] Marra G, Wood SN (2011). Practical variable selection for generalized additive models. Comput Stat Data Anal.

