# Supplementary material for: Using non-parametric Bayes shrinkage to assess relationships between multiple environmental and social stressors and neonatal size and body composition in the Healthy Start cohort
Source: Environ Health. 2022 Nov 19;21:111. doi: 10.1186/s12940-022-00934-z (PMC9675112; doi:10.1186/s12940-022-00934-z)
Supplement: Supplementary file 1 — Additional file 1. Supplemental Materials. [file 12940_2022_934_MOESM1_ESM.docx]

Supplemental Materials

Using non-parametric Bayes shrinkage to assess relationships between multiple environmental and social stressors and neonatal size and body composition in the Healthy Start cohort

Sheena E. Martenies*^1,2,3^, Lauren Hoskovec^4^, Ander Wilson^4^, Brianna F. Moore^5^, Anne P. Starling^6,7^, William B. Allshouse^8^, John L. Adgate^8^, Dana Dabelea^5,6,9^, and Sheryl Magzamen^3,5^

^1^ Department of Kinesiology and Community Health, University of Illinois at Urbana-Champaign, Urbana, IL USA

^2^ Division of Nutritional Sciences, University of Illinois at Urbana-Champaign, Urbana, IL USA

^3^ Department of Environmental and Radiological Health Sciences, Colorado State University, Fort Collins, CO USA

^4^ Department of Statistics, Colorado State University, Fort Collins, CO USA

^5^ Department of Epidemiology, Colorado School of Public Health, University of Colorado Anschutz Medical Campus, Aurora, CO, USA

^6^ Lifecourse Epidemiology of Adiposity and Diabetes (LEAD Center), University of Colorado Anschutz Medical Campus, Aurora CO, USA

^7^ Department of Epidemiology, Gillings School of Global Public Health, University of North Carolina, Chapel Hill, NC USA

^8^ Department of Environmental and Occupational Health, Colorado School of Public Health, University of Colorado Anschutz Campus, Aurora, CO, USA

^9^ Department of Pediatrics, School of Medicine, University of Colorado Anschutz Medical Campus, Aurora, CO, USA

*Corresponding Author

Sheena E. Martenies

Department of Kinesiology and Community Health

University of Illinois at Urbana-Champaign

906 S Goodwin Ave

M/C 052

Urbana, IL 61801

smarte4@illinois.edu

This supplemental materials document contains:

Supplemental Text (1)

Supplemental Tables (3)

Supplemental Figures (4)

Text S1. Fitting the NPB Model

As described in the main paper, the response variable is modeled as:

$$y_{I}|\beta,\gamma,\zeta,\delta,\sigma^{2} \sim N(y_{o}+ x_{i}^{T}\beta+z_{i}^{T}\zeta+ w_{i}^{T}\gamma, \sigma^{2})$$

where $x_{i}$ is a vector of exposures, $z_{i}$ is a vector of pairwise multiplicative interactions between the exposures or the exposures and the covariates, $w_{i}$ is a vector of covariates, and$\sigma^{2}$ is the error variance. The main effects regression coefficients $\beta$ and the interaction term coefficients $\zeta$ are modeled with a Dirichlet process (DP) prior and the covariate coefficients $\gamma$ and error variance $\sigma^{2}$ are modeled with semi-conjugate priors. These priors are defined as follows:

$$\beta_{j}|D_{1}\sim D_{1}, j=1,\ldots p$$

$$D_{1}|\alpha_{1}, D_{01}\sim DP(\alpha_{1},D_{01})$$

$$D_{01}\left| \pi_{01}= \pi_{01}\delta_{0}+(1-\pi_{01} \right)G_{1}$$

$$G_{1}|\mu_{1},\phi_{1}^{2}\equiv N(\mu_{1},\phi_{1}^{2})$$

$$\mu_{1}\sim N(0, \sigma_{\mu_{1}}^{2})$$

$$\phi_{1}^{-2} \sim Gamma\left( \alpha_{\phi1},\beta_{\phi1} \right)$$

$$\pi_{01}\sim Beta(\alpha_{\pi},\beta_{\pi})$$

$$\alpha_{1}=Gamma(\alpha_{\alpha1},\beta_{\alpha1})$$

$$\zeta_{jk}| D_{2}\sim D_{2},j=1,\ldots p-1, k=j+1, \ldots p$$

$$D_{2}| \alpha_{02}D_{02}\sim DP(\alpha_{2},D_{2})$$

$$D_{02}\left| \pi_{02}= \pi_{02}\delta_{0}+(1-\pi_{02} \right)G_{2}$$

$$G_{2}|\mu_{2},\phi_{2}^{2}\equiv N(\mu_{2},\phi_{2}^{2})$$

$$\mu_{2}\sim N(0, \sigma_{\mu_{2}}^{2})$$

$$\phi_{2}^{-2} \sim Gamma\left( \alpha_{\phi2},\beta_{\phi2} \right)$$

$$\pi_{02}\sim Beta(\alpha_{\pi2},\beta_{\pi2})$$

$$\alpha_{2}=Gamma(\alpha_{\alpha2},\beta_{\alpha2})$$

$$\gamma_{0}\sim N(\mu_{0},\kappa_{0}^{2})$$

$$\gamma\sim N(\mu_{\gamma},\kappa^{2}I)$$

$$\sigma^{-2}\sim Gamma(\alpha_{\sigma},\beta_{\sigma})$$

Prior to fitting the NPB model, we identified the set of prior distributions for the model. We set $\alpha_{\pi}=\beta_{\pi}=$ 5 so that the mass of the beta distribution was between 0.4 and 0.6. We also set $\alpha_{\phi_{1}}=$ 10 and $\sigma_{\mu_{1}}^{-2}=$ 10. For all other hyperparameters, we used the default settings as provided in the package “mmpack” (Hoskovec, 2019).

The NPB model was implemented using 5000 iterations, with 2500 discarded as burn-in.

Table S1. Results of single exposure linear regression models adjusted for all individual level covariates.^1^ Results are presented for a 1 SD increase in each exposure. Results where the confidence interval does not contain zero are bolded

|  |  | Birth Weight (g) | Adiposity (%Fat Mass) |
| --- | --- | --- | --- |
| Environmental Exposure | SD^2^ | Beta (95% CI) | Beta (95% CI) |
| Mean PM_2.5_ (μg/m^3^) | 0.6 | 8.7 (-34.8, 52.1) | -0.04 (-0.40, 0.33) |
| Mean O_3_ (ppb) | 3.1 | -41.9 (-104.3, 20.5) | -0.24 (-0.77, 0.28) |
| Mean temperature (°F) | 4.8 | -22.1 (-83.9, 39.6) | -0.16 (-0.67, 0.36) |
| Tree cover (%) | 3.1 | 12.9 (-27.6, 53.3) | 0.11 (-0.22, 0.45) |
| Impervious surfaces (%) | 13.3 | -16.4 (-54.4, 21.6) | -0.12 (-0.44, 0.19) |
| AADT (vehicles per day per km^2^) | 8203 | 10.2 (-25.1, 45.4) | 0.18 (-0.11, 0.47) |
| Distance to TRI sites (km) | 2.6 | 12.9 (-28.9, 54.8) | 0.08 (-0.26, 0.43) |
| Distance to NPL sites (km) | 3.3 | 19.8 (-17.1, 56.7) | 0.13 (-0.18, 0.44) |
| Distance to waste sites (km) | 2.3 | **39.7 (4.1, 75.4)** | **0.36 (0.07, 0.66)** |
| Distance to major emitters (km) | 3.2 | 16.1 (-19, 51.3) | 0.13 (-0.16, 0.42) |
| Distance to CAFOs (km) | 6.8 | -27.5 (-177.1, 122.2) | 0.02 (-1.19, 1.23) |
| Distance to mines or wells (km) | 2.1 | -13.3 (-57.7, 31.1) | -0.07 (-0.44, 0.30) |
| Social Exposures |  |  |  |
| CVD hospitalizations (n per 10,000) | 45.2 | -19.4 (-55.3, 16.5) | -0.24 (-0.54, 0.05) |
| Respiratory hospitalizations (n per 10,000) | 33.0 | -24.3 (-60.1, 11.5) | -0.25 (-0.55, 0.05) |
| Violent crimes (n per 1,000) | 6.3 | -4.2 (-37.7, 29.4) | -0.19 (-0.47, 0.09) |
| Property crimes (n per 1,000) | 36.0 | -11.1 (-44.4, 22.2) | **-0.31 (-0.59, -0.04)** |
| Less than HS diploma (%) | 12.7 | -18.6 (-58.5, 21.4) | -0.20 (-0.53, 0.13) |
| Unemployment (%) | 5.0 | **-40.9 (-76.8, -4.9)** | -0.28 (-0.58, 0.02) |
| Households speaking limited English (%) | 10.9 | -14.5 (-52.1, 23.1) | -0.03 (-0.33, 0.28) |
| Households in poverty (%) | 8.3 | -16.2 (-53.2, 20.8) | -0.25 (-0.55, 0.06) |
| Persons of color (%) | 22.9 | -7.1 (-50, 35.8) | -0.07 (-0.42, 0.28) |
| ^1^ Models are adjusted for: maternal race/ethnicity, maternal educational attainment, maternal pre-pregnancy BMI, maternal age at delivery, maternal smoking during pregnancy, second-hand smoke exposure during pregnancy, mean perceived stress scale score across pregnancy, mean postnatal depression score across pregnancy, season of conception, year of conception, longitude, latitude, and the interaction between longitude and latitude. Models of adiposity are also adjusted for the number of days between delivery and PEA POD measurements.  ^2^ Effect estimates are reported for a 1 standard deviation increase in each exposure variable | | | |

Table S2. Results from the sensitivity analysis exploring alternative values for the mass concentration hyperparameter in the NPB models of birth weight

| Gamma distribution parameters | $\alpha_{\alpha1}=1 ,\beta_{\alpha1}=1$  $\alpha_{\alpha2}=1 ,\beta_{\alpha2}=1$ | | | $\alpha_{\alpha1}=2 ,\beta_{\alpha1}=2$  $\alpha_{\alpha2}=2 ,\beta_{\alpha2}=2$ | | $\alpha_{\alpha1}=4 ,\beta_{\alpha1}=2$  $\alpha_{\alpha2}=4 ,\beta_{\alpha2}=2$ | | $\alpha_{\alpha1}=1 ,\beta_{\alpha1}=0.5$  $\alpha_{\alpha2}=1 ,\beta_{\alpha2}=0.5$ | | |
| --- | --- | --- | --- | --- | --- | --- | --- | --- | --- | --- |
| Variable | Posterior Mean^a^  (95% CI) | PIP | Posterior Mean^a^  (95% CI) | | PIP | Posterior Mean^a^  (95% CI) | PIP | | Posterior Mean^a^  (95% CI) | PIP |
| Environmental Exposures |  |  |  | |  |  |  | |  |  |
| Mean PM_2.5_ (μg/m^3^) | -3.0 (-33.5, 6.1) | 0.35 | -11.4 (-73.3, 3.4) | | 0.47 | -4.2 (-43.3, 7.1) | 0.36 | | -3.9 (-45.7, 7.3) | 0.34 |
| Mean O_3_ (ppb) | -6.0 (-60.2, 5.8) | 0.38 | -13.5 (-93.4, 3.9) | | 0.48 | -6.1 (-62.4, 6.9) | 0.37 | | -8.8 (-84.2, 6.5) | 0.40 |
| Mean temperature (°F) | 0.3 (-16.3, 21.5) | 0.32 | -2.5 (-35.9, 11.2) | | 0.31 | -1.2 (-22.5, 14.6) | 0.31 | | 8.9 (-18.7, 186.1) | 0.34 |
| Mean O_3_ × Mean temperature | -156.3 (-207.7,  -116.4) | 1.00 | -195.1 (-251.6,  -125.7) | | 1.00 | -161.6 (-222.1, -118.5) | 1.00 | | -164.1 (-250.9,  -114.9) | 1.00 |
| Tree cover (%) | -0.8 (-15.2, 8.7) | 0.28 | -0.8 (-15.5, 8.4) | | 0.25 | -0.8 (-15.1, 9.3) | 0.28 | | -0.5 (-13.5, 10.8) | 0.28 |
| Impervious surfaces (%) | -1.0 (-14.8, 7.8) | 0.29 | -1.3 (-17.5, 5.8) | | 0.25 | -1 (-16.7, 7.6) | 0.27 | | -1.0 (-16.1, 8.1) | 0.27 |
| AADT (vehicles per day-km^2^) | 0.6 (-8.3, 17.2) | 0.26 | 0.7 (-8.6, 17.8) | | 0.22 | 0.5 (-9.4, 16.7) | 0.25 | | 0.8 (-8.2, 19.1) | 0.25 |
| Distance to TRI sites (km) | -1.1 (-17.3, 9.4) | 0.31 | -1.0 (-16.9, 7.6) | | 0.25 | -1.5 (-19.2, 8.6) | 0.30 | | -1.4 (-18.2, 8.6) | 0.29 |
| Distance to NPL sites (km) | -0.2 (-12.3, 11.8) | 0.28 | -0.2 (-11.9, 12.5) | | 0.24 | -0.3 (-13.5, 11.3) | 0.26 | | -0.2 (-11.2, 11.6) | 0.24 |
| Distance to waste sites (km) | 3.3 (-7.6, 41.2) | 0.33 | 2.2 (-8.2, 35.4) | | 0.27 | 2.9 (-8, 39.6) | 0.30 | | 3.1 (-7.3, 40.5) | 0.31 |
| Distance to major emitters (km) | 0.2 (-10.5, 12.9) | 0.27 | 0.0 (-10.5, 11.8) | | 0.22 | 0.1 (-10.7, 13.8) | 0.24 | | 0.2 (-10.6, 15.1) | 0.26 |
| Distance to CAFOs (km) | -2.3 (-28.9, 12.8) | 0.35 | -4.3 (-56.8, 11.2) | | 0.34 | -2.4 (-32.7, 14.3) | 0.33 | | -2.4 (-33.4, 12.5) | 0.32 |
| Distance to mines or wells (km) | -2.0 (-22.6, 8.4) | 0.33 | -3.3 (-34.4, 5.9) | | 0.32 | -2.3 (-25.6, 7.5) | 0.32 | | -1.9 (-24.4, 9.1) | 0.31 |
| Social Exposures |  |  |  | |  |  |  | |  |  |
| CVD hospitalizations (n per 10,000) | -1.2 (-16.2, 6.0) | 0.29 | -1.7 (-20.8, 5.5) | | 0.27 | -1.5 (-17.6, 7.0) | 0.28 | | -1.5 (-19.8, 7.8) | 0.30 |
| Resp. hospitalizations (n per 10,000) | -2.4 (-25.2, 4.4) | 0.32 | -2.9 (-28.1, 3.3) | | 0.31 | -2.5 (-25.1, 5.8) | 0.32 | | -2.9 (-30.5, 4.1) | 0.32 |
| Violent crimes (n per 1,000) | -0.3 (-12.8, 11.8) | 0.28 | -0.7 (-13.7, 8.4) | | 0.24 | -0.4 (-12.2, 11.2) | 0.27 | | -0.4 (-12.2, 9.3) | 0.24 |
| Property crimes (n per 1,000) | -1.5 (-18.4, 7.2) | 0.32 | -1.8 (-21.6, 5.3) | | 0.28 | -1.6 (-18.4, 6) | 0.29 | | -1.5 (-18.9, 6.5) | 0.28 |
| Less than HS diploma (%) | -1.3 (-17.9, 10.3) | 0.31 | -2.0 (-23.3, 5.6) | | 0.28 | -1.6 (-20.3, 7.6) | 0.30 | | -1.7 (-19.5, 8.5) | 0.29 |
| Unemployment (%) | -7.7 (-60.5, 2.4) | 0.44 | -6.7 (-50.2, 2.2) | | 0.39 | -7.0 (-55.9, 2.3) | 0.41 | | -7.1 (-51.7, 2.1) | 0.44 |
| Households speaking limited English (%) | -1.2 (-17.2, 7.9) | 0.29 | -1.3 (-18.5, 6.7) | | 0.25 | -1.5 (-17.3, 5.5) | 0.27 | | -1.3 (-17, 6.9) | 0.28 |
| Households in poverty (%) | -1.0 (-15.3, 8.9) | 0.29 | -1.0 (-16.5, 8.3) | | 0.26 | -1.1 (-16.4, 8.1) | 0.26 | | -1.3 (-16.8, 7.4) | 0.27 |
| Persons of color (%) | -0.4 (-15.7, 14.7) | 0.30 | -0.5 (-15.5, 11.1) | | 0.24 | -0.9 (-17.3, 10.5) | 0.27 | | -0.2 (-13.5, 12) | 0.25 |
| ^a^ Models are adjusted for: maternal race/ethnicity, maternal educational attainment, maternal pre-pregnancy BMI, maternal age at delivery, maternal smoking during pregnancy, second-hand smoke exposure during pregnancy, mean perceived stress scale score across pregnancy, mean postnatal depression score across pregnancy, season of conception, year of conception, longitude, latitude, and the interaction between longitude and latitude.  ^b^ Models of adiposity are also adjusted for the number of days between delivery and PEA POD measurements.  ^c^ Effect estimates are reported for a 1 standard deviation increase in each exposure variable | | | | | | | | | | |

Table S3. Results from the sensitivity analysis exploring alternative values for the mass concentration hyperparameter in the NPB models of adiposity

| Gamma distribution parameters | $\alpha_{\alpha1}=1 ,\beta_{\alpha1}=1$  $\alpha_{\alpha2}=1 ,\beta_{\alpha2}=1$ | | | $\alpha_{\alpha1}=2 ,\beta_{\alpha1}=2$  $\alpha_{\alpha2}=2 ,\beta_{\alpha2}=2$ | | $\alpha_{\alpha1}=4 ,\beta_{\alpha1}=2$  $\alpha_{\alpha2}=4 ,\beta_{\alpha2}=2$ | | $\alpha_{\alpha1}=1 ,\beta_{\alpha1}=0.5$  $\alpha_{\alpha2}=1 ,\beta_{\alpha2}=0.5$ | | |
| --- | --- | --- | --- | --- | --- | --- | --- | --- | --- | --- |
| Variable | Posterior Mean^a,b^  (95% CI) | PIP | Posterior Mean^a,b^  (95% CI) | | PIP | Posterior Mean^a,b^  (95% CI) | PIP | | Posterior Mean^a,b^  (95% CI) | PIP |
| Environmental Exposures |  |  |  | |  |  |  | |  |  |
| Mean PM_2.5_ (μg/m^3^) | -0.01 (-0.15, 0.10) | 0.27 | -0.01 (-0.14, 0.10) | | 0.29 | -0.01 (-0.16, 0.11) | 0.3 | | -0.01 (-0.16, 0.12) | 0.28 |
| Mean O_3_ (ppb) | -0.03 (-0.30, 0.10) | 0.35 | -0.03 (-0.24, 0.08) | | 0.35 | -0.03 (-0.27, 0.07) | 0.34 | | -0.03 (-0.28, 0.08) | 0.33 |
| Mean temperature (°F) | -0.02 (-0.22, 0.10) | 0.32 | -0.01 (-0.20, 0.11) | | 0.31 | -0.02 (-0.23, 0.11) | 0.33 | | -0.01 (-0.19, 0.10) | 0.29 |
| Mean O_3_ × Mean temperature | 0.00 (-0.07, 0.00) | 0.03 | -0.01 (-0.09, 0.00) | | 0.05 | 0.00 (-0.06, 0.00) | 0.03 | | -0.01 (-0.11, 0.00) | 0.05 |
| Tree cover (%) | -0.01 (-0.15, 0.10) | 0.27 | 0.00 (-0.13, 0.11) | | 0.27 | -0.01 (-0.16, 0.12) | 0.28 | | 0.00 (-0.13, 0.13) | 0.26 |
| Impervious surfaces (%) | -0.01 (-0.15, 0.10) | 0.27 | -0.01 (-0.15, 0.08) | | 0.28 | -0.01 (-0.16, 0.06) | 0.28 | | -0.01 (-0.16, 0.08) | 0.27 |
| AADT (vehicles per day-km^2^) | 0.01 (-0.08, 0.20) | 0.26 | 0.01 (-0.09, 0.22) | | 0.28 | 0.01 (-0.08, 0.23) | 0.26 | | 0.02 (-0.07, 0.24) | 0.25 |
| Distance to TRI sites (km) | -0.01 (-0.18, 0.10) | 0.29 | -0.01 (-0.15, 0.10) | | 0.3 | -0.01 (-0.16, 0.11) | 0.29 | | -0.01 (-0.16, 0.10) | 0.27 |
| Distance to NPL sites (km) | 0.00 (-0.13, 0.10) | 0.26 | 0.00 (-0.14, 0.13) | | 0.27 | 0.00 (-0.14, 0.14) | 0.27 | | 0.00 (-0.12, 0.14) | 0.25 |
| Distance to waste sites (km) | 0.03 (-0.09, 0.40) | 0.30 | 0.04 (-0.07, 0.43) | | 0.33 | 0.04 (-0.07, 0.43) | 0.33 | | 0.04 (-0.06, 0.40) | 0.31 |
| Distance to major emitters (km) | 0.00 (-0.09, 0.20) | 0.24 | 0.01 (-0.09, 0.19) | | 0.27 | 0.01 (-0.10, 0.19) | 0.27 | | 0.01 (-0.09, 0.21) | 0.25 |
| Distance to CAFOs (km) | -0.02 (-0.26, 0.20) | 0.33 | -0.01 (-0.26, 0.19) | | 0.36 | -0.02 (-0.27, 0.17) | 0.36 | | -0.02 (-0.25, 0.17) | 0.3 |
| Distance to mines or wells (km) | -0.03 (-0.28, 0.00) | 0.36 | -0.03 (-0.26, 0.07) | | 0.36 | -0.03 (-0.27, 0.05) | 0.36 | | -0.03 (-0.27, 0.05) | 0.32 |
| Social Exposures |  |  |  | |  |  |  | |  |  |
| CVD hospitalizations (n per 10,000) | -0.02 (-0.22, 0.00) | 0.32 | -0.02 (-0.20, 0.06) | | 0.33 | -0.02 (-0.23, 0.07) | 0.35 | | -0.02 (-0.19, 0.08) | 0.3 |
| Resp. hospitalizations (n per 10,000) | -0.03 (-0.23, 0.00) | 0.32 | -0.02 (-0.23, 0.05) | | 0.33 | -0.02 (-0.22, 0.05) | 0.32 | | -0.02 (-0.22, 0.06) | 0.3 |
| Violent crimes (n per 1,000) | -0.01 (-0.15, 0.10) | 0.29 | -0.01 (-0.15, 0.07) | | 0.29 | -0.01 (-0.16, 0.09) | 0.3 | | -0.01 (-0.16, 0.09) | 0.27 |
| Property crimes (n per 1,000) | -0.07 (-0.39, 0.00) | 0.48 | -0.06 (-0.39, 0.03) | | 0.47 | -0.07 (-0.39, 0.02) | 0.5 | | -0.06 (-0.40, 0.03) | 0.44 |
| Less than HS diploma (%) | -0.02 (-0.20, 0.10) | 0.31 | -0.02 (-0.19, 0.09) | | 0.32 | -0.02 (-0.23, 0.07) | 0.3 | | -0.02 (-0.22, 0.07) | 0.28 |
| Unemployment (%) | -0.04 (-0.31, 0.00) | 0.37 | -0.04 (-0.32, 0.05) | | 0.39 | -0.04 (-0.34, 0.04) | 0.38 | | -0.04 (-0.33, 0.04) | 0.34 |
| Households speaking limited English (%) | 0.00 (-0.12, 0.20) | 0.27 | 0.01 (-0.10, 0.19) | | 0.28 | 0.01 (-0.11, 0.23) | 0.28 | | 0.01 (-0.11, 0.22) | 0.26 |
| Households in poverty (%) | -0.02 (-0.23, 0.00) | 0.31 | -0.02 (-0.21, 0.06) | | 0.33 | -0.03 (-0.25, 0.04) | 0.33 | | -0.02 (-0.23, 0.05) | 0.28 |
| Persons of color (%) | 0.00 (-0.14, 0.10) | 0.27 | 0.00 (-0.13, 0.16) | | 0.29 | 0.00 (-0.13, 0.14) | 0.27 | | 0.00 (-0.13, 0.14) | 0.26 |
| ^a^ Models are adjusted for: maternal race/ethnicity, maternal educational attainment, maternal pre-pregnancy BMI, maternal age at delivery, maternal smoking during pregnancy, second-hand smoke exposure during pregnancy, mean perceived stress scale score across pregnancy, mean postnatal depression score across pregnancy, season of conception, year of conception, longitude, latitude, and the interaction between longitude and latitude.  ^b^ Models of adiposity are also adjusted for the number of days between delivery and PEA POD measurements.  ^c^ Effect estimates are reported for a 1 standard deviation increase in each exposure variable | | | | | | | | | | |


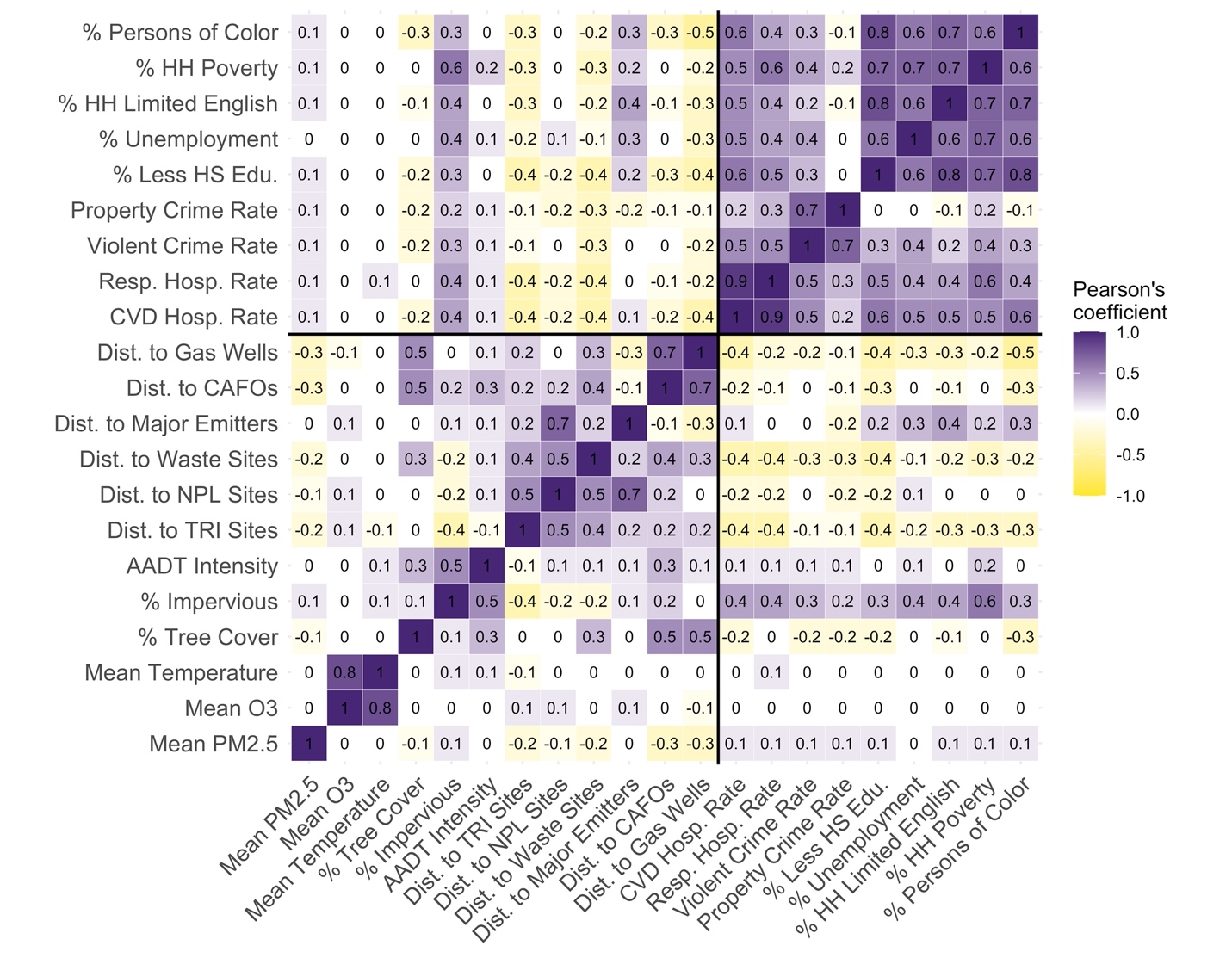


Figure S1. Correlations between exposure variables


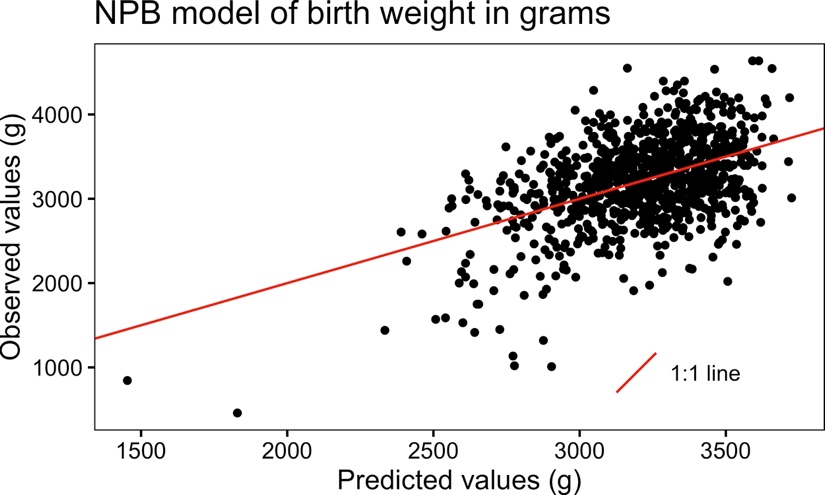


Figure S2. Scatter plot showing the relationship between birth weight predicted by the NPB model and the observed birth weights in the Healthy Start cohort.


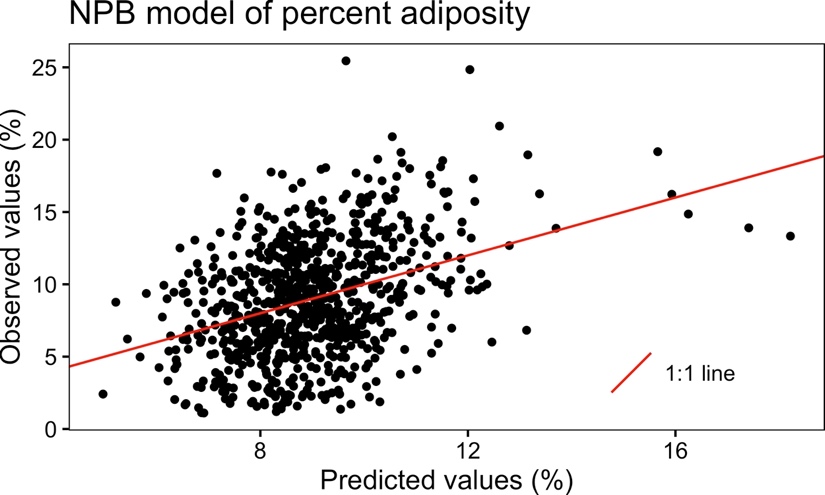


Figure S3. Scatter plot showing the relationship between adiposity predicted by the NPB model and the observed adiposity in the Healthy Start cohort.


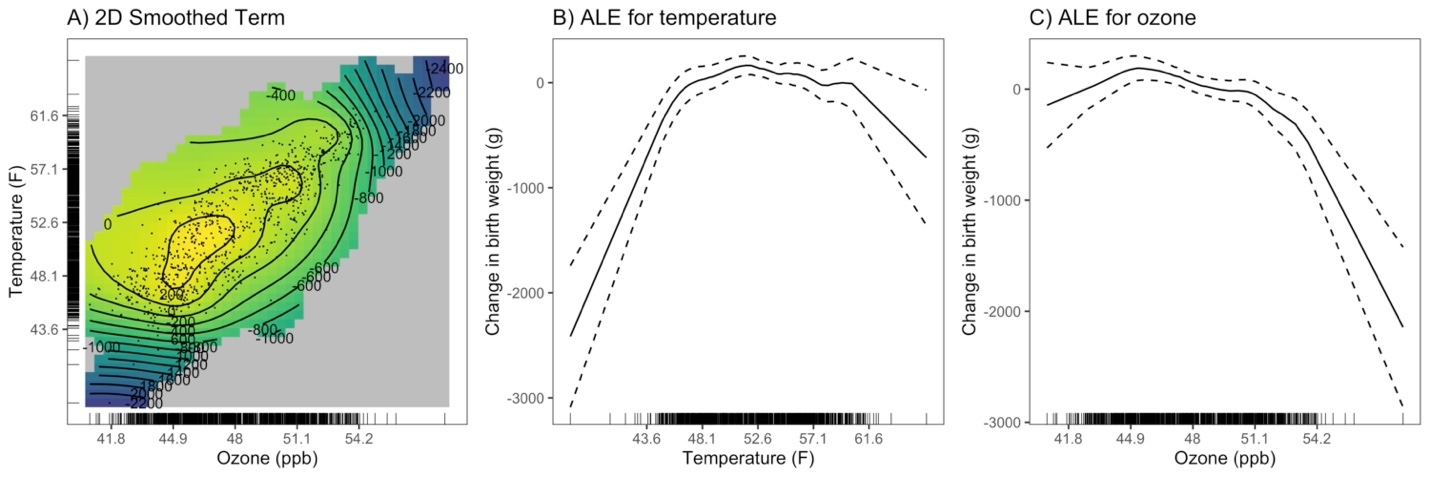


Figure S4. Exposure response curve for the 2D smoothed term for ozone and temperature in the generalized additive model (A) and accumulated local effects (ALE) plots showing the effect of temperature (B) and ozone (C) on birth weight.


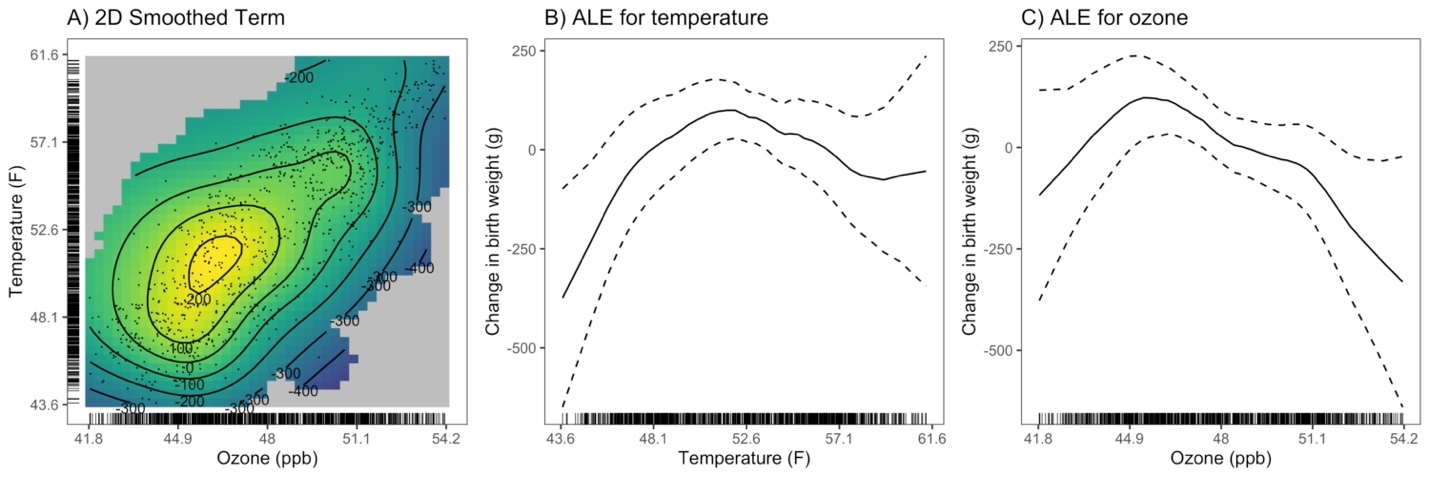


Figure S5: Sensitivity analysis restricting the data in the GAM to the middle 95% of temperature and ozone observations. Plots show the exposure-response curve for the 2D smoothed term for ozone and temperature in the generalized additive model (A) and accumulated local effects (ALE) plots showing the effect of temperature (B) and ozone (C) on birth weight.


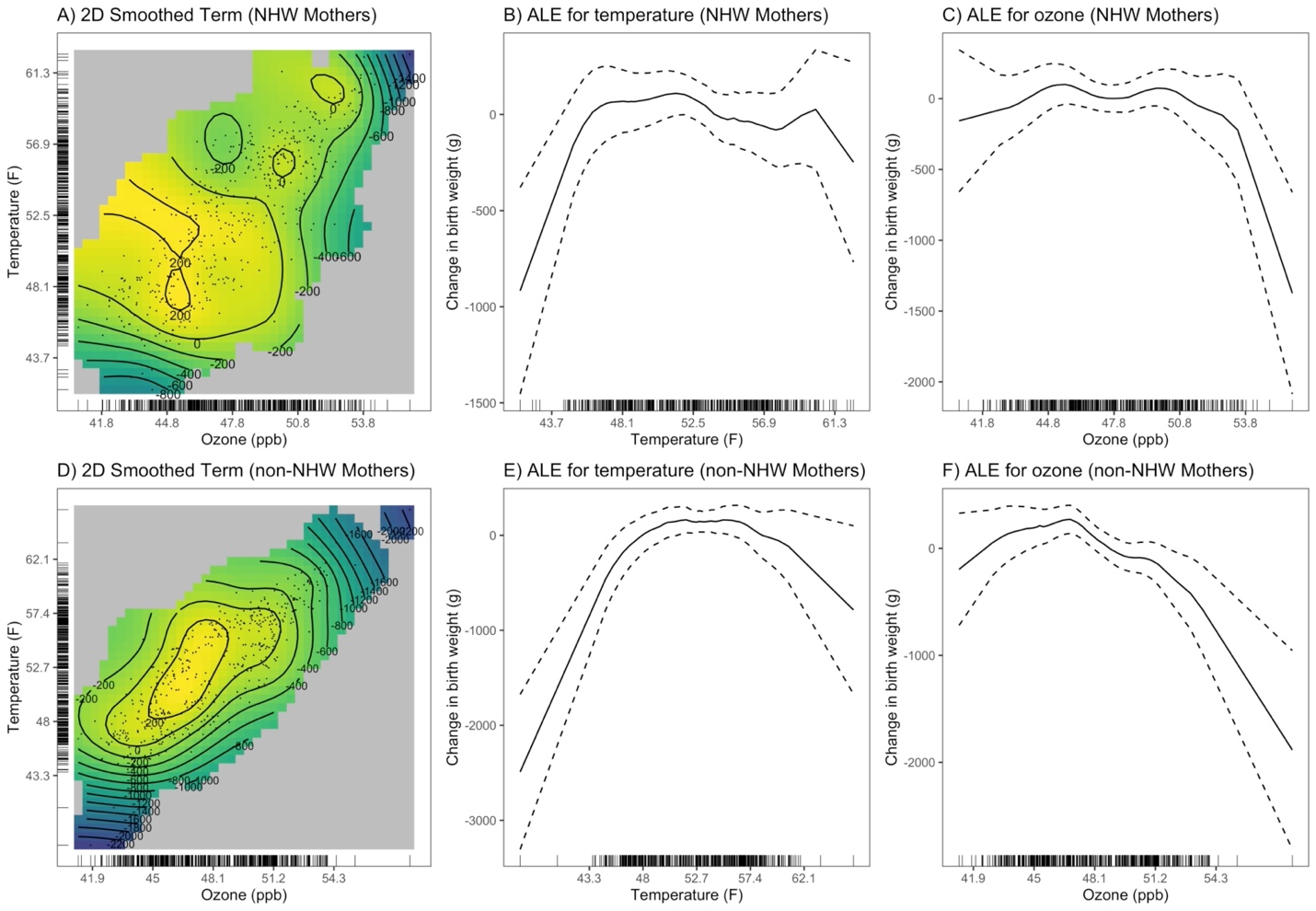


Figure S6: GAMs stratified by maternal race/ethnicity. Plots show the exposure-response curve for the 2D smoothed term for ozone and temperature in the generalized additive model (A) and accumulated local effects (ALE) plots showing the effect of temperature (B) and ozone (C) on birth weight for non-Hispanic White (NHW) mothers and the exposure response curve for the 2-D smoothed term for ozone and temperature in the generalized additive model (D) and ALE plots showing the effect of temperature (E) and ozone (F) on birth weight for mothers identifying as any race or ethnicity other than non-Hispanic White. Note: Models were fit using all available data.
